# Sites associated with Kalydeco binding on human Cystic Fibrosis Transmembrane Conductance Regulator revealed by Hydrogen/Deuterium Exchange

**DOI:** 10.1038/s41598-018-22959-6

**Published:** 2018-03-16

**Authors:** Laura J. Byrnes, Yingrong Xu, Xiayang Qiu, Justin D. Hall, Graham M. West

**Affiliations:** Structural & Molecular Sciences, Pfizer, 445 Eastern Point Road, Groton, Connecticut 06340 United States

## Abstract

Cystic Fibrosis (CF) is caused by mutations in the Cystic Fibrosis Transmembrane Conductance Regulator (CFTR). Mutations associated with CF cause loss-of-function in CFTR leading to salt imbalance in epithelial tissues. Kalydeco (also called VX-770 or ivacaftor) was approved for CF treatment in 2012 but little is known regarding the compound’s interactions with CFTR including the site of binding or mechanisms of action. In this study we use hydrogen/deuterium exchange (HDX) coupled with mass spectrometry to assess the conformational dynamics of a thermostabilized form of CFTR in apo and ligand-bound states. We observe HDX protection at a known binding site for AMPPNP and significant protection for several regions of CFTR in the presence of Kalydeco. The ligand-induced changes of CFTR in the presence of Kalydeco suggest a potential binding site.

## Introduction

Loss-of-function mutations to Cystic Fibrosis Transmembrane Conductance Regulator (CFTR) cause Cystic Fibrosis (CF), a condition affecting *ca* 1 in 2500 births^1^. CFTR is an essential part of salt homeostasis in epithelial tissues. In the lungs, loss-of-function CFTR mutations cause thicker mucus that is difficult to clear and increases susceptibility to bacterial infection^2^. Chronic infection leads to lung trauma and eventual organ failure. In addition to pulmonary effects, CF patients also suffer from pancreatic deficiency, poor nutrient absorption, and sterility in males^2^. There are 242 known CF-causing mutations in CFTR, though as many as 92% of patients have a deletion of phenylalanine 508 (ΔF_508_) on at least one allele (https://www.cftrscience.com/cftr-mutations). Common CF-causing mutations were used for a phenotypic screen that resulted in the development of two novel small molecule therapeutics, Kalydeco and Lumacaftor (also called VX-809) that aid the majority of patients^3^. Although not curative, Kalydeco and Lumacaftor are a breakthrough in CF therapy as they demonstrate defects associated with CFTR can be targeted by molecular therapeutics.

CFTR is a member of the C-subgroup of ABC transporters, which typically couple ATP hydrolysis to the transport of ligands against their concentration gradient^4^. CFTR is the only known ABC transporter that is a chloride selective ion channel, coupling ATP-binding to the gating of chloride down its electrochemical gradient. CFTR has two transmembrane domains (TMDs), two nucleotide binding domains (NBDs) and a Regulatory Domain (RD) connecting NBD1 and TMD2. RD phosphorylation (by Protein Kinase A [PKA] and other kinases^5–7^) enables channel opening. CFTR activity is also controlled by the binding of ATP, which is thought to cause dimerization of the NBDs^8^. Mutations that affect CFTR gating are known to cause CF, including the two most common, ΔF_508_ and G_551_D. Although the mechanism remains unclear, evidence suggests the CF-therapeutic Kalydeco increases channel opening by an ATP-independent mechanism after RD phosphorylation^9,10^.

Kalydeco potentiates CFTR^9–11^; however, the site of binding, and how it affects the function and stability of CFTR are unknown. In a purified liposome ion flux assay system, Eckford and colleagues showed Kalydeco could enhance phosphorylated wild type and mutant CFTR-dependent ion flux. This effect was ATP-independent, but in the wild type and ΔF_508_ the responses were additive with ATP. Interestingly, ion flux in the G_551_D mutant was not enhanced by adding nucleotide. Furthermore, Kalydeco can potentiate channels that completely lack NBD2^12,13^ as well as a number of other CF mutants with gating defects^14,15^. These have led to the hypothesis that Kalydeco may act by reducing the energy barrier to channel opening via binding somewhere in the TMDs^9^ or TMD/NBD interfaces^16^, thereby facilitating the conformational changes associated with channel opening in a mechanism independent of ATP-induced activation.

To date, there have been no reports to show the combined effect of AMPPNP and Kalydeco. In addition the CFTR literature has not yet clarified the effect that AMPPNP has on the channel, and some conflicting results exist, but in general they lead to the conclusion that this ATP analog may be inhibitory when bound to site 1 only. First, many labs have shown that AMPPNP fails to activate the channel without a small amount of ATP^17–21^. Aleksandrov *et al*. showed AMPPNP bound preferentially at site 1^22^ and Weinreich *et al*. observed slowed opening kinetics when ATP was applied with AMPPNP, implying that AMPPNP could bind to the closed state and in fact was inhibitory at site 1^23^. Zerhusen *et al*. and Mathews *et al*. noted inhibition of AMPPNP in the presence of ATP when AMPPNP was added at millimolar concentrations, presumably because it begins to out-compete ATP for site 1^24,25^. It is also interesting to note that in the crystal structure of the related bacterial ABC transporter TM287/288 the bound AMPPNP was only found at site 1, with the protein adopting a closed (inward-facing) conformation^26^. However, additional studies are needed to clarify the structural effect that AMPPNP has on CFTR.

Hydrogen/deuterium exchange (HDX) mass spectrometry is a well-established tool for characterizing protein conformational dynamics and protein-ligand binding interactions^27,28^. Integral membrane proteins present significant technical challenges for HDX, such as the production of suitable protein samples and the ability to achieve sufficient sequence coverage. Prior to this work, HDX experiments were performed on isolated NBD1 of CFTR to compare the stability of wild type versus ΔF_508_ constructs^29^ and investigate the effect of Lumacaftor binding^30^. Recently, HDX was successfully employed to compare the open and closed conformations of the bacterial ABC transporter BmrA^31^. Here we apply HDX to a full length human CFTR construct and find differential effects by two ligands on the phosphorylated form of the protein. The non-hydrolysable ATP analog AMPPNP imparts protection to exchange in the nucleotide’s binding site. We were able to detect significant protection to exchange by Kalydeco in close proximity to F_508_. AMPPNP diminishes conformational changes imparted by Kalydeco which would support AMPPNP acting as an inhibitor. This study represents the first application of HDX to characterize ligand binding interactions of a full length mammalian membrane ABC transporter. In addition this work provides a structural mechanism of action for Kalydeco’s ability to potentiate CFTR and suggests a potential binding site.

## Results

### Construct design, purification, and functional validation

**Figure 1:**
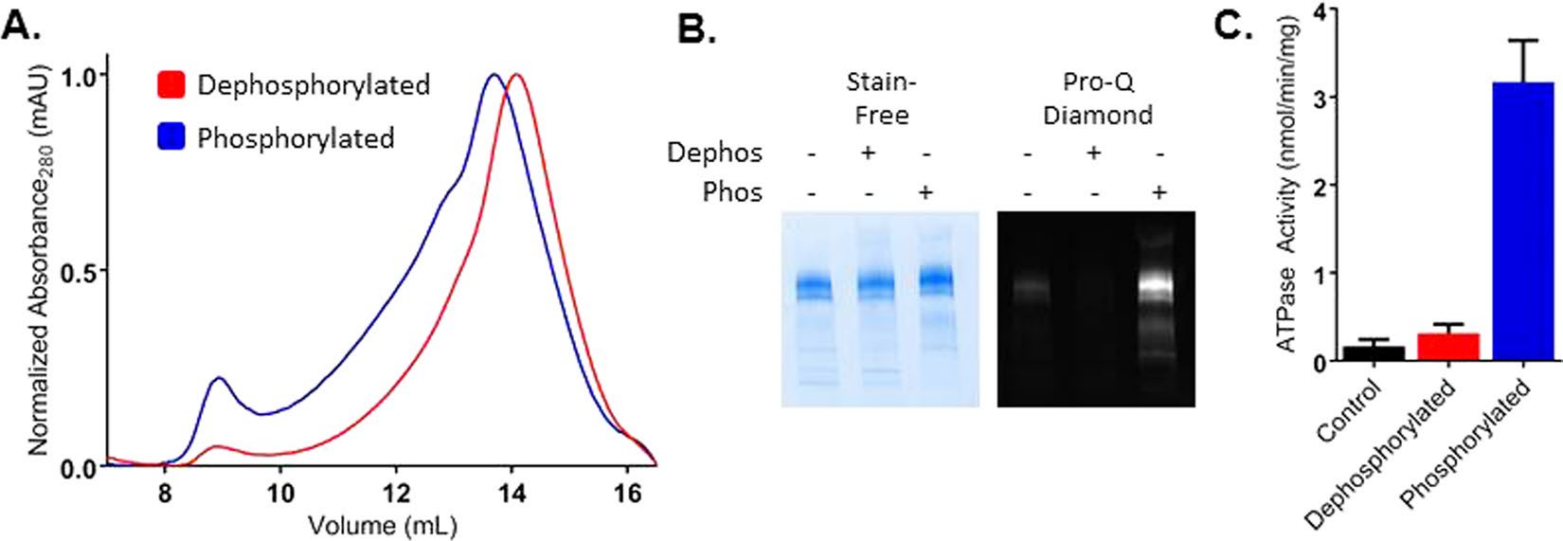
FL hCFTR^TS^ purification and activity. (**A**) Representative chromatograms for dephosphorylated (red) and PKA phosphorylated (blue) hCFTR^TS^ run over a Superose 6 10/300 SEC column as a final purification step. (**B**) hCFTR^TS^ after purification (no phosphatase or kinase treatment in lane (1), post λPP treatment in lane (2), and post PKA treatment in lane (3) was run on stain-free Bio-rad SDS-PAGE and imaged (left image). The same gel was then stained using Pro-Q Diamond Phosphoprotein Stain to confirm phosphorylation state after λPP and PKA treatment. The full-length gel images are shown in Figure S1B and C. (**C**) ATPase activity of purified dephosphorylated (red) and phosphorylated (blue) hCFTR^TS^ at 0.5 µM in the presence of 5 mM ATP was measured using the PK/LDH coupled enzyme assay.

Full length human CFTR is notoriously difficult to purify at concentrations required for biophysical analysis, in part due to the low basal expression level. To circumvent this issue, we used a higher expressing thermodynamically stabilized construct that has been shown to increase CFTR expression through a 32 amino-acid deletion and 8 point mutations. These mutations have been previously characterized^32–40^ and enabled these HDX studies on active phosphorylated CFTR, rather than the non-phosphorylated form used for recent structural studies^41^. This is hereafter referred to as thermostabilized CFTR (hCFTR^TS^). With this construct we observe an approximate 5-fold increase in expression over wild type in transiently transfected HEK Expi293 cells (Figure S1). In addition to higher expression, hCFTR^TS^ could be purified to a monodisperse homogeneous product in DDM detergent (Fig. 1A), yielding between 1–1.25 mg of fully purified protein per liter of cell culture (*ca* 5–8 × 10^9^ cells). Protein purified from Expi293 cells is partially phosphorylated, but can be dephosphorylated with Lambda Phosphatase treatment and subsequently phosphorylated with PKA (Fig. 1B) on 8 serine residues in the RD (pS_660_, pS_700_, pS_712_, pS_737_, pS_753_, pS_768_, pS_795_, and pS_813_, wild-type hCFTR sequence numbering) that were confirmed by mass spectrometry (Figure S2). After purification, an ATPase assay verified that purified CFTR retained ATPase activity. Consistent with previous reports^42,43^, phosphorylation stimulated ATPase activity of the detergent-solubilized protein approximately 10-fold (3.17 nmol/min/mg *vs* 0.31 nmol/min/mg), but was about 45-fold slower compared to activity measured in the presence of lipids or digitonin^41,44^ (Fig. 1C).

### Effects of Ligand Binding on Thermal Stability

The SYPRO Orange (SO) dye assay was used to measure the melting temperature (T_m_) of hCFTR^TS^ in the presence and absence of ligands. Since this dye detects denatured protein by associating with hydrophobic surfaces it cannot normally be used for determining the T_m_ of membrane proteins because of the necessary presence of detergent, which increases the SO background fluorescence. By decreasing the detergent concentration to the CMC (0.006% in 150 mM NaCl, Anatrace website), the background fluorescence was small enough to clearly observe the unfolding transition (Figure S3A) over the background. In addition, it was observed that Kalydeco significantly increased the SO background fluorescence, and therefore had to be kept at low micromolar concentrations.

The stability of our hCFTR^TS^ construct was higher (>3–10 °C) compared to published results for wild type^42^, consistent with the observed increase in expression (Figures S1A and S3B). Dephosphorylated CFTR was less stable than the phosphorylated form by 8 °C, and both showed a slight but not statistically significant stabilization (*ca* 1 °C) in the presence of 1 mM AMPPNP (Figure S3B). Interestingly, the presence of Kalydeco caused a decrease (−5 °C for dephosphorylated, −9 °C for phosphorylated CFTR at 10 µM) in thermal stability that was not significantly affected by the addition of AMPPNP. The presence of 10 µM Kalydeco also decreased the temperature at which CFTR first forms aggregates (in the absence of extrinsic dyes) as monitored by static light scattering (SLS) at 266 and 473 nm (Figure S3C and D). Addition of CFTR corrector Lumacaftor caused an increase (3 °C for dephosphorylated, 5 °C for phosphorylated CFTR at 10 µM) in thermal stability that was not significantly affected by addition of AMPPNP.

### HDX sequence coverage and receptor conformational mobility

HDX measurements were performed on the apo and ligand-bound (ADP, AMPPNP, Kalydeco, or AMPPNP + Kalydeco) PKA phosphorylated full length hCFTR^TS^ and on apo and ligand-bound (Lumacaftor) dephosphorylated hCFTR^TS^. HDX analysis covered 60% of the sequence (Figure S4A) at all exchange time points for the apo protein and all protein-ligand complexes. Individual domain sequence coverage was much higher for the more solvent accessible RD (84%) compared to the hydrophobic TMDs (43%) (Figure S4B). Low sequence coverage for the hydrophobic TMDs is typical as peptides from these regions show poor chromatographic performance due to their hydrophobicity, protease occlusion by the micelle environment and a higher frequency of pepsin cleavage sites leading to decreased LC-MS detection^45^.

**Figure 2:**
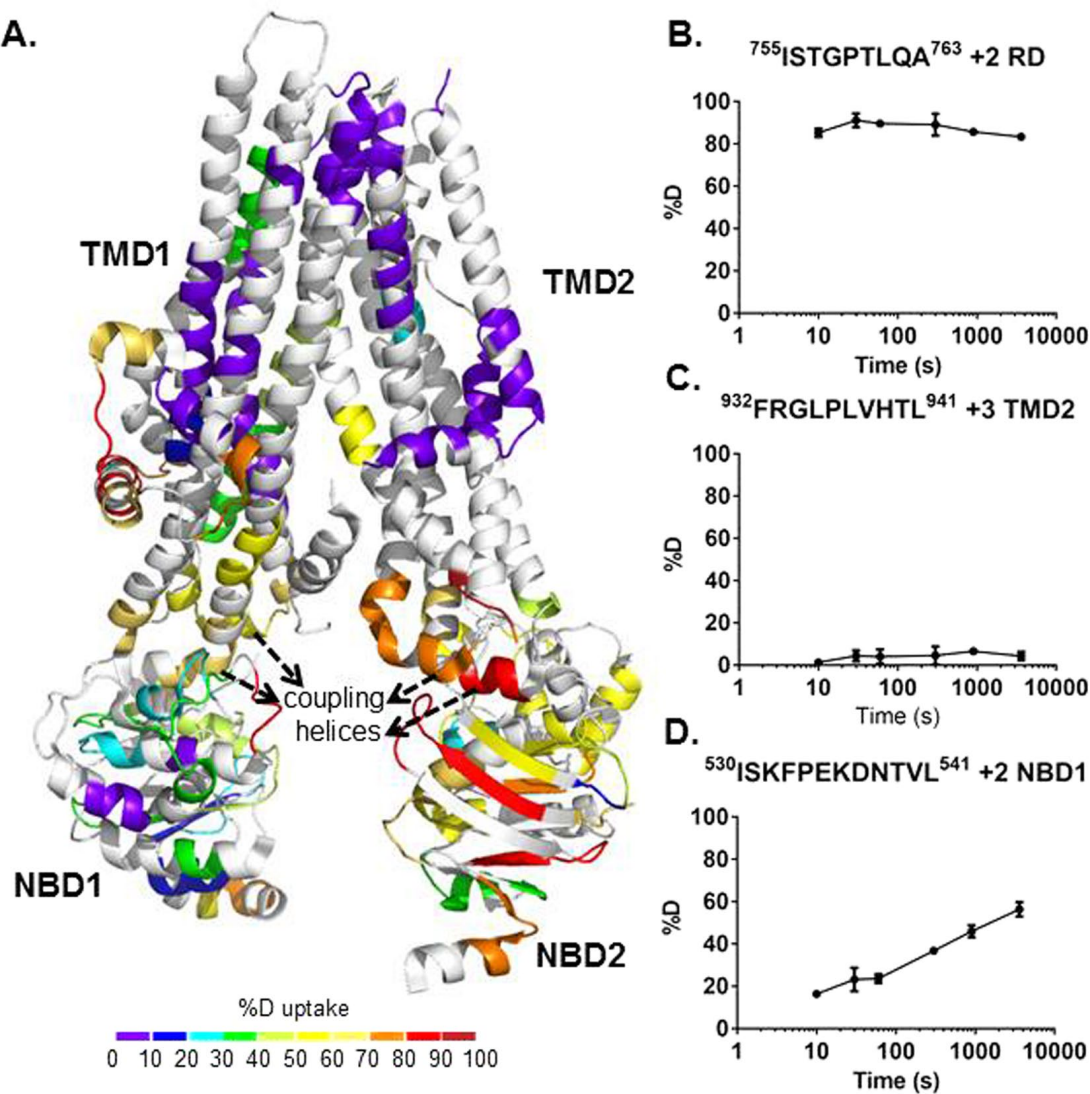
HDX profile of apo hCFTR^TS^. (**A**) HDX data from the heat map (see Figure S5) is indicated on the 3D cryo-EM structure of human CFTR (PDB 5UAK). The R domain is not shown in the cyro-EM structure since it was not fully resolved. As shown in the key, a color gradient is used to represent average deuterium uptake values across all 6 time points ranging from 10 s to 3600 s. White indicates regions that were not detected for every replicate at every time point in the HDX experiment. (**B**–**D**) Representative deuterium uptake time-course curves are shown for peptides from RD (**B**), TMD2 (**C**) and NBD1 (**D**). Data represent the means ± SD (n = 3). Charge state of the detected peptide ion is also noted.

The deuterium exchange of apo phosphorylated hCFTR^TS^ was monitored over six time points ranging from 10 s to 1 h (Figure S5). The rate of amide backbone hydrogen exchange is affected by hydrogen bonding, with α-helices and β-sheets generally having a lower extent of deuterium exchange than unstructured regions. Consistent with these expectations we observed the intrinsically disordered RD to have higher average deuterium uptake (80%) than the more ordered TMDs (25%). The average deuterium uptake values across all time points for the apo state were mapped onto the 3.9 Å cryo-EM structure of human CFTR (Fig. 2A)^41^. The HDX data shows good correlation with the structure where a greater extent of exchange was observed for the unresolved RD and relatively low exchange in the TMDs (Fig. 2). However, one notable deviation from this trend is observed in the increased exchange at NBD2 relative to NBD1 which indicates NBD1 may be a more thermodynamically stable domain.

### AMPPNP NBD1 Binding Site Characterization by HDX

**Figure 3:**
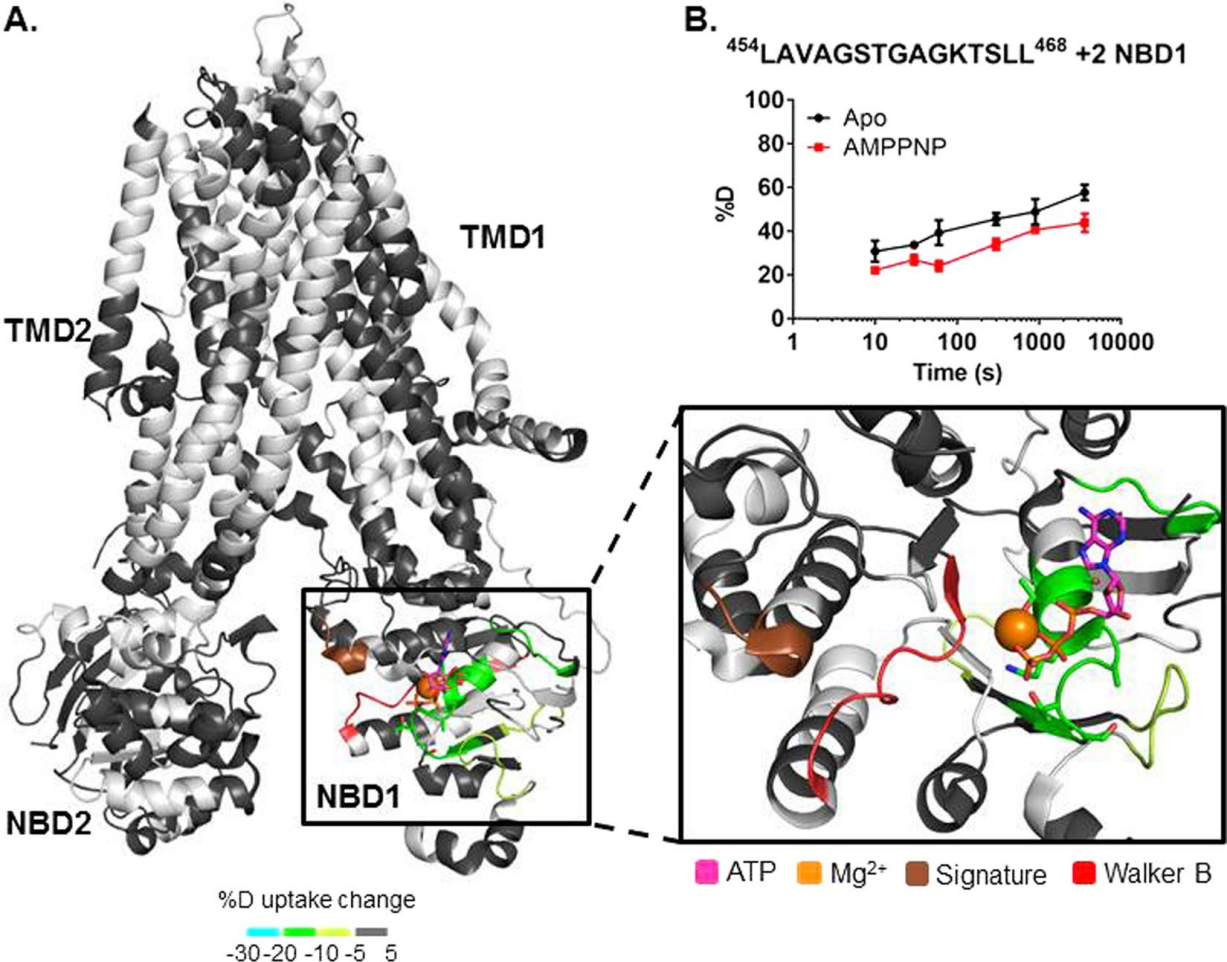
Stabilization of hCFTR^TS^ upon AMPPNP binding. (**A**) HDX perturbation data mapped to the cryo-EM structure (PDB 5UAK). As shown in the key, a color gradient is used to represent the average deuterium uptake differences across all 6 time points between the apo and AMPPNP bound states of hCFTR^TS^. White indicates regions that were not detected for every replicate at every time point in the HDX experiments. NBD1 is rotated and zoomed in to show the stabilized peptides. Conserved motifs are highlighted as follows: Walker A sidechains (sticks), signature sequence (brown), and Walker B (red). Estimated location of ATP (magenta, sticks) and magnesium (orange, ball) are shown based on alignment with NBD1 crystal structure (2BBO). (**B**) Deuterium uptake time-course plots of a peptide from the Walker A motif in both apo and AMPPNP bound states. Data represent the means ± SD (n = 3). Charge state of the peptide is also noted.

In the consensus model for ATP-dependent activation of CFTR the NBDs form a head-to-tail dimer which induces channel opening after RD phosphorylation and ATP binding^8,46^. One ATP molecule binds at each of two sites in the NBD dimer interface (site 1 and site 2), which are predominantly formed by NBD1 and NBD2. Binding at two sites has also been shown for ADP^23^. Both sites have interactions contributed from multiple motifs including the phosphate-interacting Walker A motif (Fig. 3A)^29^, the magnesium-interacting Walker B, and the ABC signature sequence (LSGGQ motif)^8^. The two ATP-binding sites exhibit distinct binding and hydrolysis behavior; site 1 has higher affinities for ATP and AMPPNP than site 2 and is unable to hydrolyze ATP due to mutations in the NBD1 Walker B motif and NBD2 signature sequence^22,47^. AMPPNP caused protection to deuterium exchange for peptides at site 1, with the greatest protection for residues 456–468 (wild type hCFTR sequence numbering) of the Walker A motif (Fig. 3B, Figure S6). ADP, the hydrolyzed product of ATP, caused protection to exchange in the same regions of CFTR as AMPPNP (Figures S7 and S8). No protection was observed around site 2 for either nucleotide which is consistent with NBD1 binding AMPPNP tighter than NBD2^22^.

### Kalydeco causes HDX protection of the TMDs

**Figure 4:**
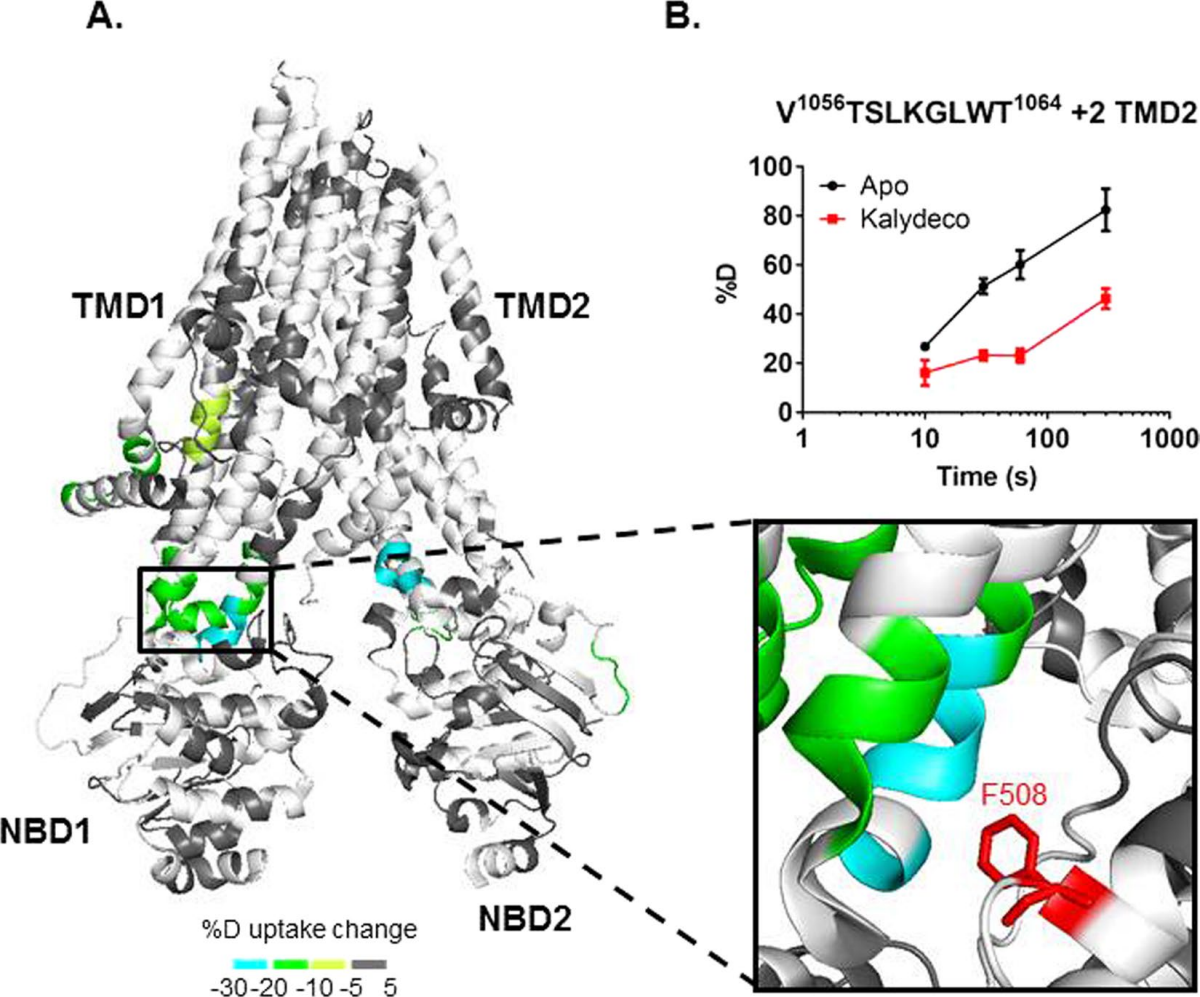
hCFTR^TS^ conformational changes upon Kalydeco binding. (**A**) HDX perturbation data mapped to the cryo-EM structure (PDB 5UAK). As shown in the key, a color gradient is used to represent the average deuterium uptake differences across all 6 time points between the apo and Kalydeco bound states of hCFTR^TS^. White indicates regions that were not detected for every replicate at every time point in the HDX experiments. The ICL4 region was zoomed in to show the peptides stabilized by Kalydeco binding. F_508_ was highlighted in red and represented as sticks. (**B**) Deuterium uptake time-course curves of the TMD2 peptide which showed protection upon ligand binding. Data represent the means ± SD (n = 2–3). Charge state of the peptide ion is also noted.

Kalydeco increases channel activity for wild type and 34 mutant CFTR forms carrying CF-causing mutations including the most common mutations, ΔF_508_ and G_551_D^3,9,10,14,15^. Activation of CFTR by Kalydeco requires PKA phosphorylation, and is both ATP-independent and additive to the normal ATP-dependent mechanism common to ABC transporters^10^. Although Kalydeco can activate purified CFTR, suggesting the drug directly binds CFTR, its binding site is unknown. The additivity of Kalydeco and ATP supports distinct activation mechanisms and therefore non-overlapping binding sites^10^. To investigate regions affected by the Kalydeco-dependent mode of activation and establish potential Kalydeco binding sites, we compared the deuterium uptake values of apo and Kalydeco-bound hCFTR^TS^ (Fig. 4A, Figure S9). Regions showing protection to deuterium exchange include a short segment of the N-terminus (63–72), residues from the intracellular loops (ICLs) (162–180, 967–973, 1055–1064 and 1070–1074), TMD1 (146–152), and NBD2 (1271–1274 and 1333–1337). The greatest protection was located in the TMD2/NBD1 interface (1055–1064, and 1070–1074) and the TMD2/NBD2 interface (967–973) with 20–30% protection to deuterium exchange compared to apo hCFTR^TS^ (Fig. 4A). Interestingly, this region is in direct contact with F_508_, the amino acid whose deletion leads to disease for the majority of CF patients. We also performed HDX of dephosphorylated CFTR in the presence of Lumacaftor, but did not detect any evidence for a conformational change compared to the apo state (Figure S10).

### AMPPNP reduces Kalydeco’s protection of the TMDs

**Figure 5:**
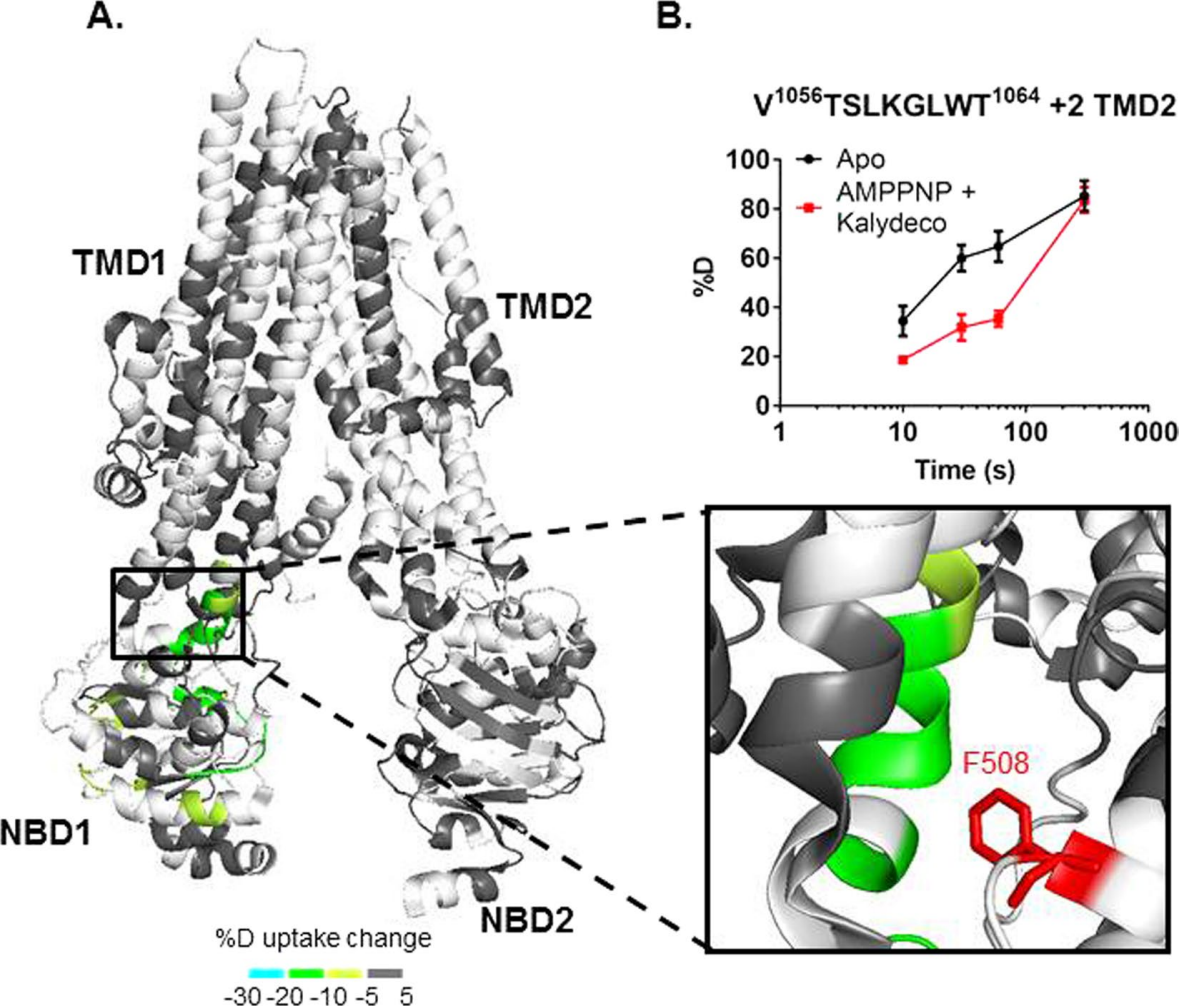
hCFTR^TS^ conformational changes when both AMPPNP and Kalydeco were bound. (**A**) HDX perturbation data mapped to the cryo-EM structure (PDB 5UAK). As shown in the key, a color gradient is used to represent the average deuterium uptake differences across all 6 time points between the apo and ligand bound states of hCFTR^TS^. White indicates regions that were not detected for every replicate at every time point in the HDX experiments. The ICL4 region was zoomed in to show the protected peptides. F_508_ was highlighted in red and represented as sticks. (**B**) Deuterium uptake time-course curves of the TMD2 peptide which showed protection upon ligand binding. Data represent the means ± SD (n = 2–3). Charge state of the peptide ion is also noted.

Compared to Kalydeco alone, addition of AMPPNP to Kalydeco-bound hCFTR^TS^ causes a reduction in the number of protected TMD peptides (from 10 to 2) and the average protection of these peptides (from 18% to 12%) (Fig. 5, Figure S11). While AMPPNP reduces, or completely eliminates, regions protected by Kalydeco alone, it also restores the majority of NBD1 protection previously observed for AMPPNP only. The Kalydeco alone and AMPPNP + Kalydeco conditions both retain protection at residues 1055–1064, which are located at the TMD2/NBD1 interface and in close proximity to F_508_ (Figs 4 and 5). The reduction in protection suggests AMPPNP may be decreasing the potentiation effect of Kalydeco and is discussed further below.

## Discussion

Efforts to isolate full length hCFTR for biophysical characterization have been hindered by low expression even in mammalian cell systems. Purification of hCFTR in quantities suitable for both T_m_ measurements and HDX was achieved by combining previously identified mutations and deletions proven to enhance CFTR expression and stability. This stabilized construct enables future biophysical studies that require milligram quantities of protein.

Thermal denaturation experiments show destabilization of the hCFTR^TS^ construct by Kalydeco (Figure S3B). In contrast, the HDX exchange experiments only showed protection to exchange in a few regions. This could indicate that thermodynamic destabilization primarily affects regions of the hCFTR^TS^ sequence that were not covered in the HDX experiment, or occur through disruption of side chain interactions. These T_m_ changes in the presence of Kalydeco support prior evidence for direct interaction with the purified protein^10,11,48^. Although Kalydeco’s effect on the thermal stability of purified CFTR has not been reported, it has been shown that chronic administration diminishes functional expression of wild type and ΔF_508_ CFTR even in combination with Lumacaftor^49,50^, which may be a result of destabilization. Ligand binding that causes destabilization in unfolding transitions have been reported^51–54^, and can occur with ligands that have an affinity for the unfolded state of the protein^55^. Alternatively, the lipophilicity of Kalydeco may cause destabilization of the detergent micelles themselves, which in turn would destabilize the protein. Cholon *et al*. postulates Kalydeco may increase conformational flexibility of the channel, allowing gating mutants such as G_551_D to access the open state. Conformational flexibility could entropically promote an unfolding transition, a phenomenon observed in numerous protein systems including CFTR^25,35,51,56,57^. This would suggest that any CFTR potentiator with this mechanism of activation may result in apparent thermal destabilization. In addition, we tested the CFTR corrector Lumacaftor for its effect on hCFTR^TS^ thermal stability and observed a compound-dependent increase in thermal stability, consistent with its function as a chaperone. A recent publication showed direct binding and thermal destabilization of NBD1 by Lumacaftor^54^. Unfortunately, the difference between NBD1 and full length CFTR makes this discrepancy hard to reconcile, though the authors of the NBD1 study speculate Lumacaftor may have allosteric effects in the full-length protein that result in stabilization, which would not be observed in NBD1 alone^58,59^.

The use of the DDM-stabilized hCFTR^TS^ enabled 60% overall sequence coverage, with 84% for the soluble RD. HDX data were mapped onto a 3.9 Å cryo-EM structure of hCFTR^41^, which is dephosphorylated while the HDX data uses the PKA-phosphorylated form. Despite this difference there is good agreement between the HDX data and the secondary structure for the resolved domains of the cryo-EM structure. Relatively low deuterium exchange was observed in regions with a high degree of secondary structure, such as the hydrophobic core of the TMDs. The highest deuterium exchange occurred in the RD. This would suggest the RD is highly dynamic, which is consistent with the inability of cryo-EM to resolve the RD due to intrinsic disorder (Fig. 2). Interestingly, NBD1 demonstrated lower deuterium incorporation compared to NBD2 (Fig. 2A), indicating NBD2 is more dynamic in solution than NBD1, which is consistent with lower stability and known challenges in NBD2 purification^58^. In addition, the majority (7 out of 8) of the thermostabilizing mutations are in NBD1, which may artificially rigidify hCFTR^TS^ NBD1 compared to native NBD1. The four intracellular coupling helices that connect the TM segments and are positioned in parallel to the plane of the membrane demonstrated rapid deuterium exchange rates (Fig. 2A). Cysteine cross-linking experiments suggest the coupling helices are responsible for both vertical (e.g. TMD1-NBD1) and orthogonal (e.g. TMD2-NBD1) interactions between TMDs and NBDs^60^. Higher deuterium incorporation in the coupling helices would be consistent with a dynamic interactive network between domains at this region.

The nucleotide binding experiments showed the greatest protection for peptides in ATP-binding site 1. These results suggest HDX is suitable for detecting direct ligand-binding interactions for full length CFTR. Interestingly, no protection was observed for site 2, suggesting AMPPNP may not be a strong binder at this site, which is consistent with prior reports for AMPPNP at site 2^22^. Furthermore, it has been shown that CFTR is not active at the concentration of AMPPNP tested here (400 µM) without ATP also present. These results therefore support previous studies that AMPPNP alone is not an activator at these concentrations though it does appear to bind at site 1^17-21^. It is unclear if the lack of CFTR activity previously reported is due to the apparent lack of binding at site 2, or if AMPPNP binding at site 1 is actually inhibitory, or both. These data support a model for AMPPNP-bound CFTR where the channel remains closed and AMPPNP is only present at site 1 ([AMPPNP] ≤400 µM).

The region with the largest decrease in deuterium uptake in the presence of Kalydeco is in ICL4 of the ball and socket joint^61,62^. This could indicate a compound binding site and this region has been previously proposed as a CFTR site for rational drug design^62^. Interestingly, F_508_ makes van der Waals interactions with residues from ICL4 and TM11 in this region. The ΔF_508_ deletion is the most common CF-causing mutation. Kalydeco is effective against this mutation and the HDX protection at ICL4 provides a structural molecular mechanism of action for potentiation of CFTR ΔF_508_. Given the proximity of ICL4 to F_508_ Kalydeco may cause protection to HDX in this region as well. Unfortunately an ion signal for the peptic peptide in this region was not reproducibly observed in these HDX experiments. ICL4 resides between ICL1 and F_508_ and it is interesting to note that ICL1 also shows protection to HDX in the presence of Kalydeco, albeit to a lower extent which could indicate allosteric stabilization in a region with close proximity to the potential binding site. ICL1 has also been reported to modulate CFTR channel activity via interactions with NBD1^63^. A number of mutational studies show this ball and socket joint region is involved in interdomain communication between NBD1 and the TMDs^16,60,64,65^. Cysteine cross-linking experiments revealed interactions between F_508_ and several aromatic residues from ICL4, including F_1068_, Y_1073_ and F_1074_^65^. Deletion of F_508_ would disrupt such interactions, therefore destabilizing the ICL4/NBD1 interface and potentially the whole channel. Stabilization of ICL4/NBD1 by HDX upon Kalydeco binding provides a mechanism of action to account for the drug’s ability to increase channel activity of ΔF_508_. The HDX data with Kalydeco is also suggestive of a potential binding site for the drug though it is important to note that regions protected in an HDX experiment can also indicate allosteric conformational changes induced by compound binding.

Protection to exchange was also observed at two other regions. One of these regions was at ICL3 in the TMD2/NBD2 interface (Fig. 4A). A peptide ion from ICL3 (residues 967–973) showed a significant decrease in deuterium exchange. ICL3 is adjacent to K_978_, a site known to cause ATP-independent, constitutive activity when mutated to cysteine, serine, or proline^66^. Both the human homology model and the phosphorylated zebra-fish structure of the open CFTR conformation shows these residues may make new contacts across the pore with ICL4^67^ (Figures S12 and S13), and the HDX data here provide additional evidence for a conformational change associated with CFTR activation in the ICL3 region. In addition to the TMD/NBD interfaces, Kalydeco also induced protection to exchange in the lasso motif at residues 63–72. Several mutations that cause CF are located in this region and the lasso motif has been linked to ion conductance^15,68^. In addition, the lasso motif interacts with membrane trafficking proteins^69,70^ and may provide a mechanism for Kalydeco to influence protein-protein interactions. No evidence of a conformational change on dephosphorylated CFTR was observed in the presence of Lumacaftor by HDX (Figure S10). This absence of a conformational change could be due to a protein-ligand complex predominantly formed through sidechain interactions which are not detectable in the HDX experiment or may require a construct with impaired stability compared to this thermostabilized construct.

To date, there have been no reports to show the combined effect of AMPPNP and Kalydeco. The HDX data with both AMPPNP and Kalydeco showed that AMPPNP-interacting peptides remained protected although most of the regions that were protected by Kalydeco alone showed reduced or no protection (Figure S14). This result could suggest AMPPNP bound to site 1 is inhibiting the potentiation effect of Kalydeco which would help explain previous observations for this nucleotide^17–25^. High concentrations (≥2 mM) of AMPPNP at 25 °C can lead to weak channel opening, but no opening was observed at lower AMPPNP concentrations, including concentrations similar to what were used in these HDX experiments^71,72^. Therefore, when interpreted in the context of the literature, our HDX data suggest AMPPNP can act as a site 1 binder and an inhibitor of CFTR. Under this model, binding of both compounds would be expected to shift CFTR conformation back to the closed state compared to Kalydeco alone. Allosteric conformational changes would be diminished while protection at the binding sites would remain. In fact, protection to exchange is only observed for two regions in the presence of both compounds, the TMD2/NBD1 ball and socket region and ATP-binding site 1. This would further support the ball and socket region of TMD2/NBD1 as the Kalydeco binding site.

The results discussed here illustrate the utility of HDX for determining how ligands bind to and affect the dynamic structure of CFTR in solution. HDX has been used to confirm the binding site of model CFTR ligands, AMPPNP and ADP, and identified conformational changes induced by the drug Kalydeco. This supports Kalydeco’s ability to directly bind CFTR and its effects are independent of activation by ATP^10,11^. Although HDX cannot unambiguously identify binding sites, the regions identified in this study (e.g. the ICL4/NBD1 interface) are consistent with current theories^9,16^, suggesting potential binding site locations for Kalydeco. This work also presents an HDX platform for characterizing and comparing the molecular mechanisms of compound action for future CF drug discovery programs.

## Methods

### CFTR Expression and Purification

Wild type human full length CFTR (aa 1–1480, Δ405–436) DNA was codon optimized for mammalian cell expression and synthesized by Genewiz into a pUC57 vector. CFTR was then subcloned into a modified pCDNA3.1 vector, yielding an N-terminally SUMO* tagged, C-terminally EGFP-His_6_-FLAG tagged fusion. Quikchange (Agilent) mutagenesis was used to modify the sequence to include selected thermostabilizing mutations (S_492_P, A_534_P, I_539_T, G_550_E, R_553_M, R_555_K, Q_637_R, Q_1411_D).

Thermostablized, full length human CFTR (hCFTR^TS^) was expressed in the Expi293 suspension cell line (Invitrogen) using the manufacturer’s instructions with some modifications. Cells were transfected at 2.5 × 10^6^ viable cells per milliliter with Expifectamine and 10 µg of DNA per 30 mL transfection (total) volume and grown 16–18 hours on an orbital shaker in an incubator set to 37 °C and 5% CO_2_. Enhancers 1 and 2 were subsequently added to the transfection and expression was continued for an additional 48 hours under the same conditions. Cells were harvested by centrifugation at 1850 g for 20 minutes at 4 °C, washed with ice cold PBS, and spun again. Cell pellets were then flash frozen in liquid nitrogen and stored at −80 °C until purification.

To purify hCFTR^TS^, cell pellets were thawed and dounced in hypotonic buffer (10 mM Tris-HCl pH 7.5, 1 mM EDTA, protease inhibitor cocktail) 40 times on ice. Broken cells were then centrifuged at 40,000 rpm in a 70 Ti rotor in a Beckman Ultracentrifuge for 25 minutes at 4 °C. Pelleted material was dounce homogenized in solubilization buffer (50 mM Tris-HCl pH 8, 200 mM NaCl, 2.5 mM MgCl_2_, 1 mM ATP, 10% Glycerol, 0.5 mM TCEP, 1% DDM, 0.1% CHS) 40 times on ice and then stirred for 1.5 hours at 4 °C. Solubilized membranes were then spun again for 45 minutes. Soluble material was passed over a packed 10 mL anti-FLAG M2 resin (Sigma) column connected to a Bio-rad FPLC system and washed with FLAG Wash Buffer (50 mM Tris-HCl pH 7.5, 350 mM NaCl, 2.5 mM MgCl_2_, 10% Glycerol, 0.1 mM TCEP, 0.1% DDM, 0.01% CHS) until the UV_280_ absorbance dropped and stabilized. Bound protein was eluted with 20 mL FLAG Elution buffer (50 mM Tris-HCl pH 8, 350 mM NaCl, 2.5 mM MgCl_2_, 10% Glycerol, 0.1 mM TCEP, 0.1% DDM, 0.01% CHS, 0.2 mg/ml FLAG peptide). Purified hCFTR^TS^ was treated with Lambda Phosphatase (NEB) for 30 minutes at room temperature before addition of 2 mL of Talon resin (Clontech) and incubated for an additional 30 minutes. The slurry was then poured into a 30 mL gravity column (Bio-rad) where unbound material was allowed to flow through. Talon resin with bound protein was washed with 5 column volumes of Talon Wash Buffer (50 mM Tris-HCl pH 7.5, 350 mM NaCl, 2.5 mM MgCl_2_, 10% Glycerol, 0.1 mM TCEP, 10 mM Imidazole, 0.1% DDM, 0.01% CHS) and either eluted with 3 column volumes of Talon Elution Buffer (50 mM Tris-HCl pH 7.5, 350 mM NaCl, 2.5 mM MgCl_2_, 10% Glycerol, 0.1 mM TCEP, 200 mM Imidazole, 0.1% DDM, 0.01% CHS) or treated with PKA (NEB) for 1 hour at room temperature with mixing. PKA treated, resin-bound protein was washed again with 5 column volumes of wash buffer and subsequently eluted. TEV and ULP1 proteases were then added to the eluted protein overnight at 4 °C. Cleaved protein was concentrated (Amicon, 100 kDa MWCO) and injected into a Superose 6 10/300 gel filtration column equilibrated in SEC Buffer (50 mM Tris-HCl pH 7.5, 150 mM NaCl, 2.5 mM MgCl_2_, 10% Glycerol, 0.5 mM TCEP, 0.03% DDM). Peak fractions were combined and concentrated, followed by determination of concentration via 280 nm absorbance (ε = 184,230 M^−1^ cm^−1^) before aliquoting. Aliquots were flash frozen in liquid nitrogen and stored at −80 °C until use. Protein purity was verified using Bio-Rad Mini-PROTEAN TGX Stain-Free SDS-PAGE gel analysis. Presence or absence of phosphorylation was assayed in the same gel with Pro-Q Diamond Phosphoprotein Gel Stain (Molecular Probes).

### Thermal Melting

#### Monitoring the thermal unfolding transition of hCFTRTS by Sypro Orange dye

Thermal melting temperature for hCFTR^TS^ was measured using the Sypro Orange fluorescent dye (Molecular Probes). Recombinant hCFTR^TS^ (15 µg/ml) was mixed with dye (5×) and indicated ligands in assay buffer (50 mM Tris-HCl pH 7.5, 150 mM NaCl, 5 mM MgCl_2_, 0.006% DDM). Reactions were incubated at room temperature for 10 minutes in 200 µl quartz cuvettes (Hellma) prior to fluorescence measurement. The Sypro Orange fluorescence signal was monitored every 0.5 °C at 300 nm excitation and 600 nm emission using a temperature controlled Cary Eclipse Fluorescence Spectrophotometer between 30–90 °C (bandwidth 5 nm; signal averaging time 0.2 seconds; temperature step 2 °C/minute). Curves were differentiated and fitted to a Lorenzian distribution to determine the melting temperature with each measurement performed in triplicate.

#### Monitoring the thermal aggregation of hCFTR^TS^ using static light scattering (SLS)

Thermal aggregation for hCFTR^TS^ was measured using the UNit (Unchained Labs). Recombinant hCFTR^TS^ (100 µg/ml) and indicated ligand (or vehicle) was diluted in assay buffer (50 mM Tris-HCl pH 7.5, 150 mM NaCl, 5 mM MgCl2, 0.01% DDM). Reactions were incubated at room temperature for 30 minutes prior to addition of 8.9 µL (with three replicates per condition) to the UNi quartz capillary strip. The full fluorescence spectrum (250–700 nm) and SLS signals were monitored continuously using lasers at 266 and 473 nm as the temperature was increased at a rate of 1 °C per minute between 20–85 °C. SLS signal versus temperature for each sample are plotted together in Supplementary Figure S3C and D.

### CFTR ATPase Activity Measurements

ATPase activity measurements were performed using the PK/LDH Assay following the manufacturer’s instructions in a black 384-well, low-volume, clear-bottom plate (Corning type 3544) in a total volume of 20 µl in assay buffer (25 mM Tris-HCl pH7.5, 150 mM NaCl, 5 mM MgCl_2_, 0.03% DDM). Recombinant hCFTR^TS^ at 0.5 µM was combined with 20/30 U PK/LDH, 250 µM NADH, and 2 mM PEP. The plate was incubated in the reaction mixture for 10 minutes at room temperature, after which the reaction was started by the addition of ATP (5 µM). Plates were assayed at 25 °C in a Tecan microplate reader (Infinite M1000), in which the absorbance at 340 nm was monitored every 30 seconds for 60 minutes. ADP concentration was determined using NADH absorption and initial velocities were calculated.

### HDX

#### MS/MS Sequence coverage of hCFTR^TS^

For the tandem mass spectrometry (MS/MS) sequence coverage experiment, the purified protein (25.7 µM) was diluted to 5 µM using a buffer composed of 50 mM Tris-HCl, 150 mM NaCl, 2% (v/v) glycerol, 0.03% (m/v) DDM, 2.5 mM MgCl_2_, pH 7.5 (referred to as H_2_O buffer hereafter). The buffer for HDX on-exchange experiments had the same composition except H_2_O was replaced with D_2_O (99.9%) (referred to as D_2_O buffer hereafter). A total of 5 µL of 5 µM hCFTR^TS^ protein solution was manually mixed with 20 µL of the H_2_O buffer and 25 µL of the quench solution containing 100 mM Na_2_HPO_4_, 0.02% DDM and 1% (v/v) TFA. The above 50 µL protein sample was then digested online by passing through an immobilized pepsin-coupled column (2.1 mm i.d. × 30 mm, NovaBioAssays, Woburn, MA) and the digest was subjected to high performance liquid chromatography (HPLC) analysis using a trap column (Hypersil Gold C8 5 µm packing, 10 × 1 mm drop-in guard, Thermo Fisher Scientific, San Jose, CA), an analytical column (Hypersil Gold C8 5 µm packing, 50 × 1 mm, Thermo Fisher Scientific, San Jose, CA) and 1/16” o.d. × 75 µm i.d. PEEKsil^TM^ tubing (IDEX Health & Science, Oak Harbor, WA). Sample loading, digestion, and desalting were driven by a capillary-scale HPLC pump (Agilent 1100 series, G1312A binary pump, Santa Clara, CA), while gradient elution was performed with a separate HPLC pump of the same make and model. For HPLC, buffer A was H_2_O containing 0.1% (v/v) formic acid, buffer B was acetonitrile containing 0.1% (v/v) formic acid. The flow rate was 50 µL/min with a gradient from 0.5% to 40% B over 60 min. The mobile phase for the immobilized pepsin-coupled column was buffer A and the flow rate was 50 µL/min. MS and collision-induced dissociation (CID) MS/MS spectra were acquired in positive ion mode on an Orbitrap Velos Pro hybrid ion trap-Orbitrap mass spectrometer (Thermo Fisher Scientific, San Jose, CA) equipped with an ESI source operated at capillary temperature of 250 °C and spray voltage of 4 kV. Mass spectra were acquired for m/z 300–2000 using a maximum injection time of 10 ms and automatic gain control (AGC) target value of 1 × 10^6^. MS/MS spectra were acquired using data-dependent acquisition with dynamic exclusion where the top 10 most abundant ions in each scan were selected and subjected to CID. MS/MS spectra were acquired at an activation Q value of 0.25, activation time of 30 ms, normalized collision energy of 35, isolation width of 2 m/z, AGC target value of 1 × 10^4^ and maximum injection time of 100 ms. Each MS scan consisted of 1 microscan under normal scan mode and each MS/MS scan was the average of 4 microscans under normal scan mode.

The resulting spectra were searched against the hCFTR^TS^ sequence using MASCOT (version 2.5.1, Matrix Science, London, UK) with a peptide mass tolerance of ± 10 ppm and a fragment mass tolerance of ± 0.8 Da. Peptides with an ion score of greater than 20 and mass error of less than 3 ppm were included in the peptide set used for HDX.

#### HDX analysis of hCFTR^TS^

Before each HDX experiment, 5 µM protein samples were made by diluting 16.4 µL 32 µM hCFTR^TS^ stock into 84.4 µL H_2_O buffer, then adding 2.1 µL 0.1 M Tris-HCl or 2.1 uL 20 mM AMPPNP or ADP followed by 2.1 µL DMSO or 5 mM Kalydeco. The final Kalydeco concentration was 100 µM, a 20-fold excess over hCFTR^TS^, to promote saturating conditions. The final concentrations of AMPPNP or ADP were 400 µM. The protein samples were allowed to equilibrate at 4 °C for 1 h before HDX analysis. For HDX MS experiments, 5 µL of 5 µM hCFTR^TS^ solution was mixed with 20 µL of the D_2_O buffer (final D_2_O content was 80%), or H_2_O buffer for controls, and incubated for various lengths of time at 4 °C, before being added to 25 µL quench solution. Deuterated protein was then digested online using the same procedure as described above. The pepsin column, trap column and analytical column utilized in the HDX MS experiments were the same as in the MS/MS sequence coverage experiment. HPLC was performed with a flow rate of 50 µL/min, and a gradient proceeded by a 0.5% to 10% B over 0.2 min ramp followed by a 10% to 30% B gradient over 5.3 min. MS spectra were acquired in the range of m/z 300–1700 in positive ion mode on the Orbitrap Velos Pro mass spectrometer (Thermo Fisher Scientific, San Jose, CA) equipped with an ESI source operated at capillary temperature of 250 °C and spray voltage of 4 kV. Protein and quench solutions, trap and analytical columns were kept at 4 °C inside of the chromatography refrigerator (Isotemp^TM^, Fisher Scientific, Waltham, MA). The online pepsin column was kept at 15 °C using a temperature controller (Analytical Sales & Services, Pompton Plains, NJ). HDX incubation was performed at 4 °C for 6 different time points: 10, 30, 60, 300, 900, and 3600 s. The experiments were performed at random order and the data for each on-exchange time point were obtained in 3 replicates. All HDX data were normalized to 100% D_2_O content, corrected for an estimated average deuterium recovery of 70%, and processed using the HDX WorkBench (version 3.3, Pascal B.D. *et al*., J. Am. Soc. Mass Spectrom. 2012, 23: 1512–1521).

### Data availability

The datasets generated during and/or analysed during the current study are available from the corresponding author on reasonable request.

## Electronic supplementary material

Supplementary Information
