# Supplementary material for: Sites associated with Kalydeco binding on human Cystic Fibrosis Transmembrane Conductance Regulator revealed by Hydrogen/Deuterium Exchange
Source: Sci Rep. 2018 Mar 16;8:4664. doi: 10.1038/s41598-018-22959-6 (PMC5856801; doi:10.1038/s41598-018-22959-6)
Supplement: Supplementary file 1 — Supplementary Information [file 41598_2018_22959_MOESM1_ESM.pdf]

Supplementary figures and information

Sites associated with Kalydeco binding on human Cystic Fibrosis Transmembrane Conductance Regulator revealed by Hydrogen/Deuterium Exchange

Laura J. Byrnes, Yingrong Xu, Xiayang Qiu, Justin D. Hall, Graham M. West

Figure S1

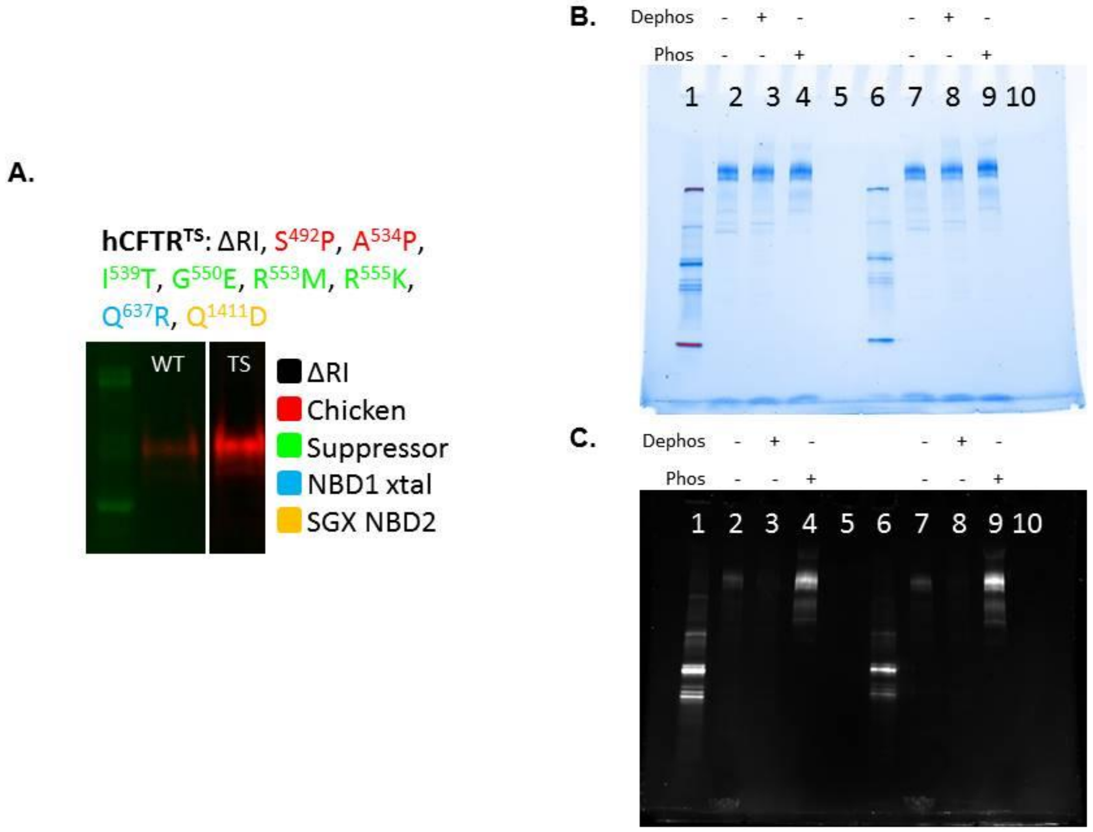

Figure S1: (A) Western blot of wild type (WT) *vs* hCFTR^{TS} (TS) constructs from detergent solubilized cell lysates (controlled by cell count). A molecular weight marker is shown in lane 1 (green). Red staining indicates binding of a FLAG-specific HRP-conjugated antibody. Constructs contain a C-terminal FLAG tag. Mutations for the hCFTR^{TS} construct are listed above the gel, colored according to mutation origin. (B) The stain-free Bio-rad SDS-PAGE imaged of the entire gel from Figure 1B. The molecular weight marker in the images is the "PeppermintStick Phosphoprotein Standard" from ThermoFisher. (C) An image of the same gel from Figure 1B with the Pro-Q Diamond Phosphoprotein Stain.

Figure S2

```
   1 MQRSPLEKASVVSKLFFSWTRPILRKGYRQRLELSDIYQIPSVDSADNLSEKLEREWDRELASKKNPKLI   70

  71 NALRRCFFWRFMFYGIFLYLGEVTKAVQPLLLGRIIASYDPDNKEERSIAIYLGIGLCLLFIVRTLLLHP  140
       |----------------------------TMD1-------------------------------------
 141 AIFGLHHIGMQMRIAMFSLIYKKTLKLSSRVLDKISIGQLVSLLSNNLNKFDEGLALAHFVWIAPLQVAL  210
     --------------------------------TMD1----------------------------------
 211 LMGLIWELLQASAFCGLGFLIVLALFQAGLGRMMMKYRDQRAGKISERLVITSEMIENIQSVKAYCWEEA  280
     --------------------------------TMD1----------------------------------
 281 MEKMIENLRQTELKLTRKAAYVRYFNSSAFFFSGFFVVFLSVLPYALIKGIILRKIFTTISFCIVLRMAV  350
     --------------------------------TMD1----------------------------------
 351 TRQFPWAVQTWYDSLGAINKIQDFLQKQEYKTLEYNLTTTEVVMENVTAFWEEGGTPVLKDINFKIERGQ  420
     ----------------TMD1-----------------|-----------------NBD1-----------
 421 LLAVAGSTGAGKTSLLMMIMGELEPSEGKIKHSGRISFCPQFSWIMPGTIKENIIFGVSYDEYRYRSVIK  490
     --------------------------------NBD1----------------------------------
 491 ACQLEEDISKFPEKDNTVLGEGGITLSEGQMAKISLARAVYKDADLYLLDSPFGYLDVLTEKEIFESCVC  560
     --------------------------------NBD1----------------------------------
 561 KLMANKTRILVTSKMEHLKKADKILILHEGSSYFYGTFSELQNLRPDFSSKLMGCDSFDQFSAERRNSIL  630
     ------------------NBD1-----------------------|-----------RE-----------
 631 TETLHRFSLEGDAPVSWTETKKQSFKQTGEFGEKRKNSILNPINSIRKFSIVQKTPLQMNGIEEDSDEPL  700
     --RE----|---------------------------RD--------------------------------
 701 ERRLSLVPDSEQGEAILPRISVISTGPTLQARRRQSVLNLMTHSVNQGQNIHRKTTASTRKVSLAPQANL  770
     --------------------------------RD------------------------------------
 771 TELDIYSRRLSQETGLEISEEINEEDLKECFFDDMESIPAVTTWNTYLRYITVHKSLIFVLIWCLVIFLA  840
     -----------------------------RD-------------------------|----TMD2-----
 841 EVAASLVVLWLLGNTPLQDKGNSTHSRNNSYAVIITSTSSYYVFYIYVGVADTLLAMGFFRGLPLVHTLI  910
     --------------------------------TMD2----------------------------------
 911 TVSKILHHKMLHSVLQAPMSTLNTLKAGGILNRFSKDIAILDDLLPLTIFDFIQLLLIVIGAIAVVAVLQ  980
     --------------------------------TMD2----------------------------------
 981 PYIFVATVPVIVAFIMLRAYFLQTSQQLKQLESEGRSPIFTHLVTSLKGLWTLRAFGRQPYFETLFHKAL  1050
     --------------------------------TMD2----------------------------------
1051 NLHTANWFLYLSTLRWFQMRIEMIFVIFFIAVTFISILTTGEGEGRVGIILTLAMNIMSTLQWAVNSSID  1120
     --------------------------------TMD2----------------------------------
1121 VDSLMRSVSRVFKFIDMPTEGKPTKSTKPYKNGQLSKVMIIENSHVKKDDIWPSGGQMTVKDLTAKYTEG  1190
     ----------------------TMD2----------------------|-------NBD2----------
1191 GNAILENISFSISPGQRVGLLGRTGSGKSTLLSAFLRLLNTEGEIQIDGVSWDSITLQQWRKAFGVIPQK  1280
     --------------------------------NBD2----------------------------------
1281 VFIFSGTFRKNLDPYEQWSDQEIWKVADEVGLRSVIEQFPGKLDFVLVDGGCVLSHGHKQLMCLARSVLS  1350
     --------------------------------NBD2----------------------------------
1351 KAKILLLDEPSAHLDPVTYQIIRRTLKQAFADCTVILCEHRIEAMLECDQFLVIEENKVRQYDSIQKLLN  1420
     --------------------------------NBD2----------------------------------
1421 ERSLFRQAISPSDRVKLFPHRNSSKCKSKPQIAALKEETEEEVQDTRL
     ----NBD2--------|
```

Figure S2: MS/MS sequence coverage map. Shown in red are sequences identified with Mascot ion score > 20 and mass accuracy < 5 ppm. An overall coverage of 75% was achieved. Phosphorylation sites with a Mascot score >20 are highlighted in green. All phosphorylation sites were confirmed by visual inspection of the MS/MS spectra. Within the sequence coverage for PKA phosphorylated $hCFTR^{TS}$, 8 phosphoserine sites were observed including pS_{660}, pS_{700}, pS_{712}, pS_{737}, pS_{753}, pS_{768}, pS_{795}, and pS_{813}. These 8 sites are in agreement with the previously reported phosphorylation sites identified by mass spectrometry or NMR (Baker et al., 2007; Neville et al., 1997; Townsend et al., 1996), though (Baker et al., 2007) identified an additional site at pS_{670} with partial phosphorylation (60%). Among the 8 phosphoserine sites, pS_{737} and pS_{768} are reported to be inhibitory (Csanady et al., 2005; Vais et al., 2004; Wilkinson et al., 1997). Sequence numbering is based on the TS construct, see Figure S4A for WT numbering alignment.

Figure S3

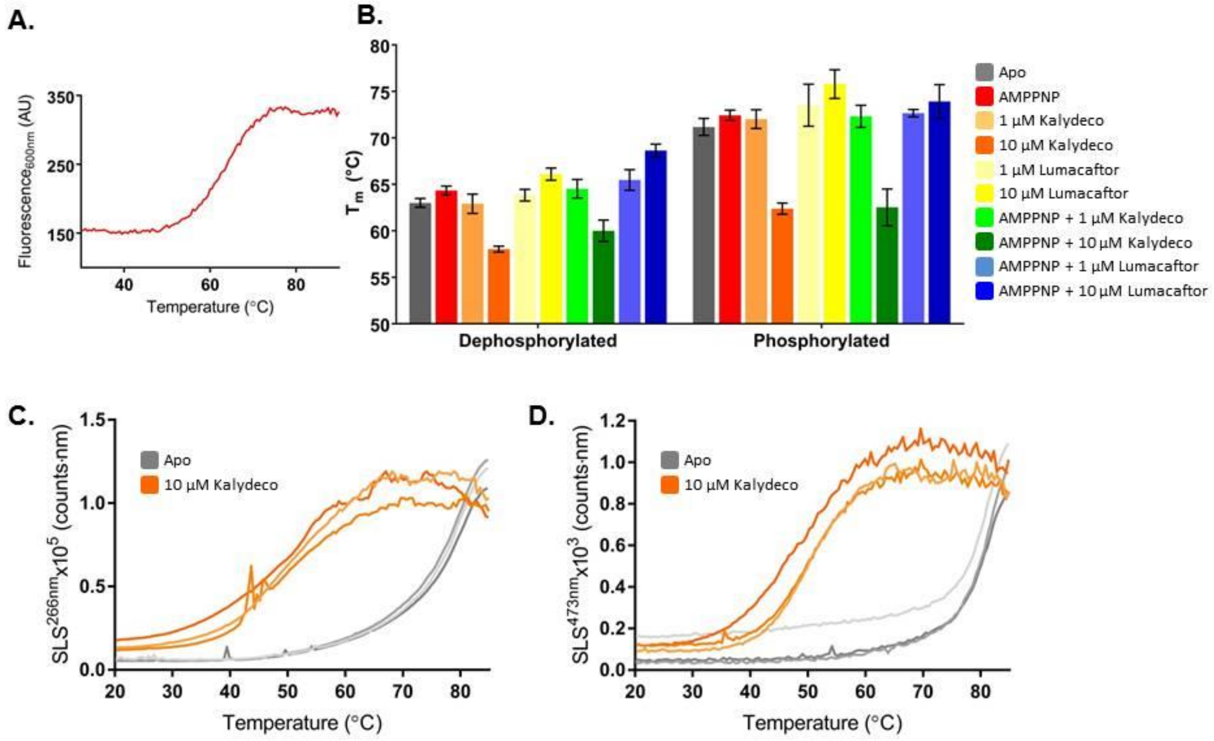

Figure S3: T_m measurements (A) Example T_m curve. (B) Average T_m values as determined by the Sypro Orange thermal shift assay for dephosphorylated and phosphorylated $hCFTR^{TS}$ in the presence of indicated ligands. All measurements were done in triplicate. (C) Static light scattering (SLS) of $hCFTR^{TS}$ from 266 nm laser in UNit instrument during a temperature ramp. SLS signal of the apo protein (shades of grey) and in the presence of 10 µM Kalydeco (shades of orange) are shown with three traces (replicates) for each. (D) As in C, but using data from 473 nm laser SLS signal.

Figure S4A

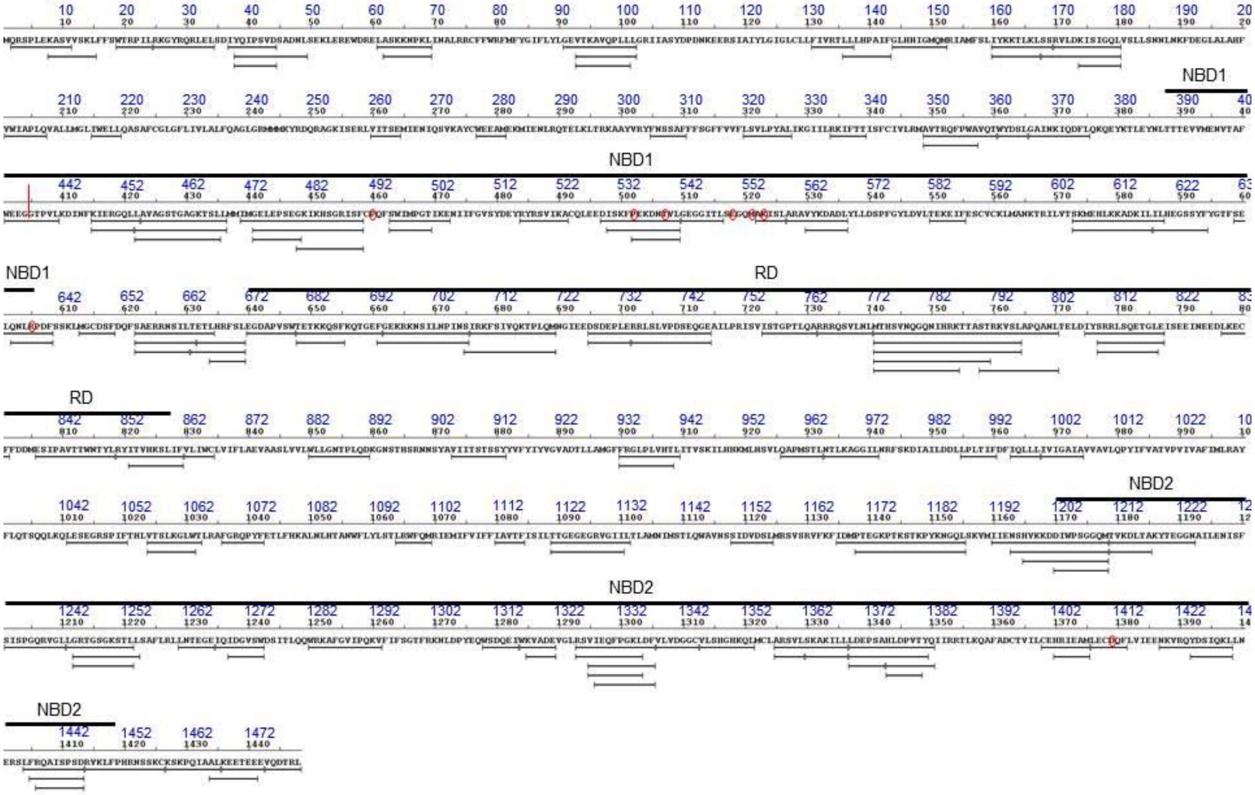

Figure S4B

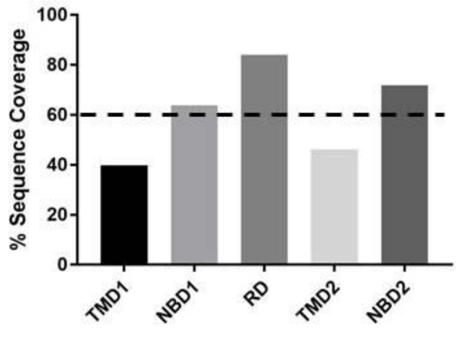

Figure S4: (A) Peptide sequence coverage map. Lines below the sequence indicate peptides that were identified by MS/MS with a Mascot ion score >20 and generated quantifiable, reproducible HDX data at all time points (10, 30, 60, 300, 900 and 3600 s) in both Apo and ligand bound forms of $hCFTR^{TS}$. Blue numbers represent sequence alignment with the wild type human CFTR. The red line indicates the location of the RI deletion and the red circles indicate points of mutation. (B) Sequence coverage of individual domains. The horizontal dashed line denotes the coverage of the full length protein (60%).

Figure S5

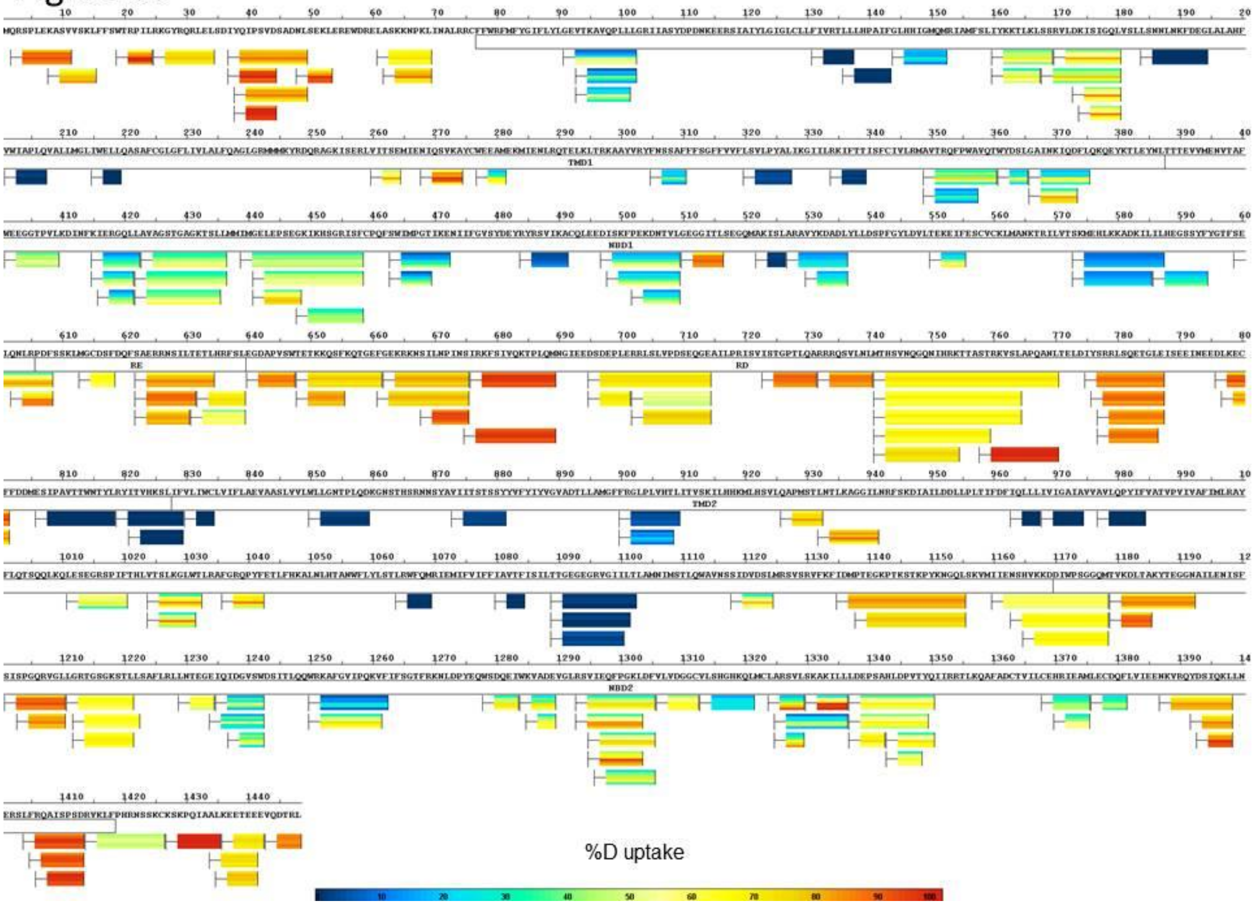

Figure S5: HDX heatmap of apo phosphorylated hCFTR^{TS} mapped to its sequence. Peptides analyzed in the HDX experiment are indicated by rectangular boxes below the hCFTR^{TS} construct sequence. Each box is subdivided into six sections representing each of the six exchange time points (from top to bottom: 10s, 30s, 1m, 5m, 15m and 1h). The percent deuterium exchange is indicated for each peptide time point according to the colored key. For each peptide, the first 2 N-terminal amino acids were excluded from the analysis due to rapid back-exchange.

Figure S6

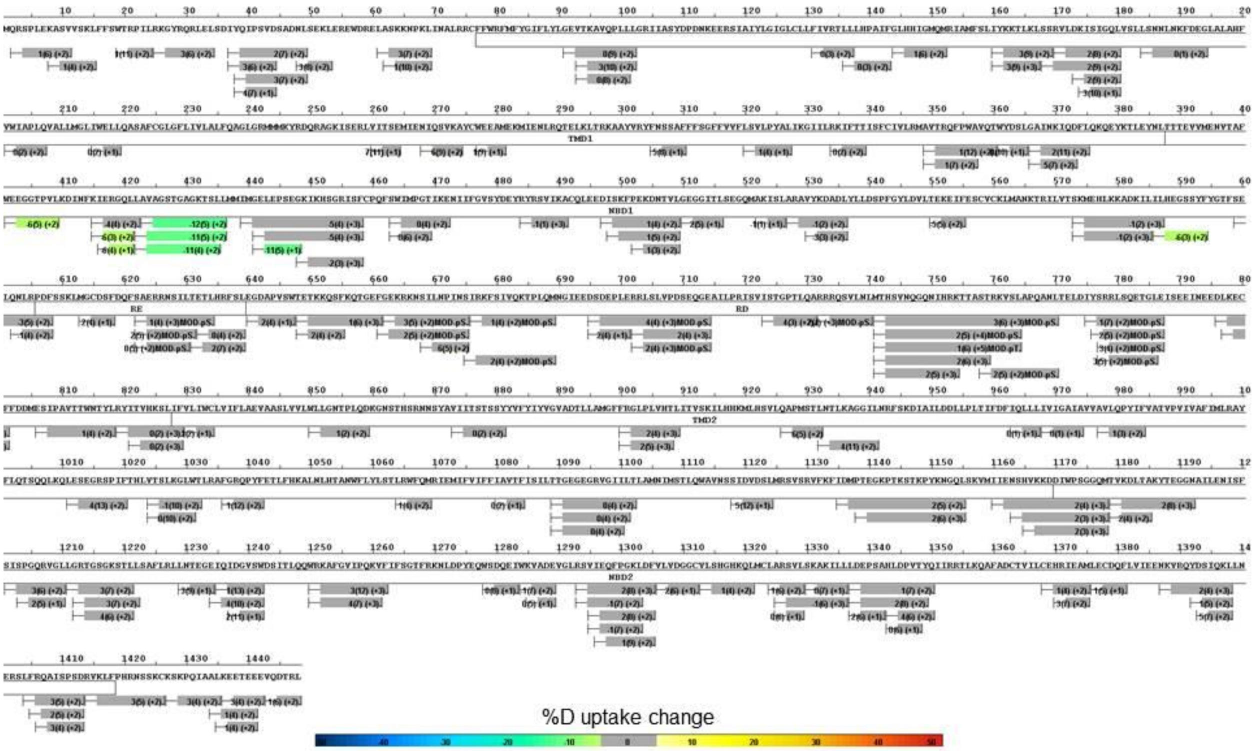

Figure S6: HDX perturbation data of AMPPNP binding mapped to hCFTR^{TS} sequence. Peptides analyzed in the HDX experiment are indicated by rectangular boxes below the hCFTR^{TS} construct sequence. The number in each box represents the change in the average deuterium uptake upon AMPPNP binding across all 6 time points. Standard error and peptide ion charge state are also noted in parenthesis. The peptide boxes are colored based on deuterium uptake differences according to the colored key. For each peptide, the first 2 N-terminal amino acids were excluded from the analysis due to rapid back-exchange.

Figure S7

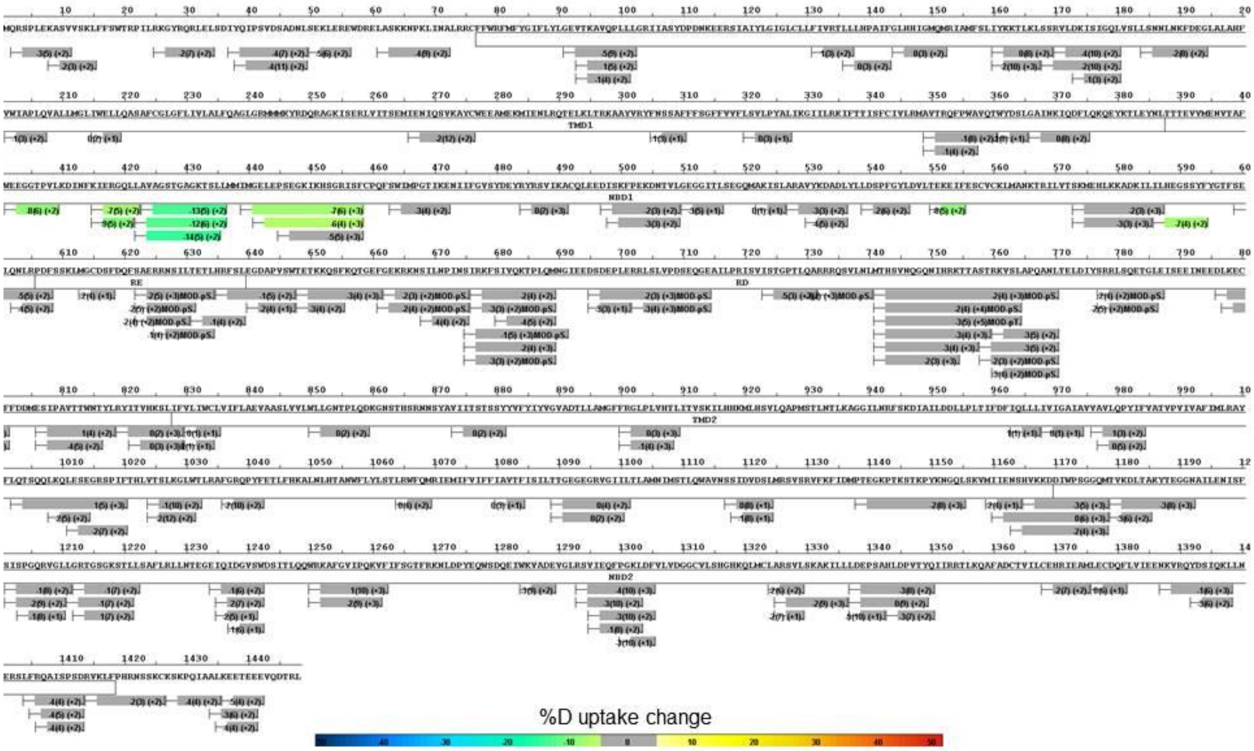

Figure S7: HDX perturbation data of ADP binding mapped to hCFTR^{TS} sequence. Peptides analyzed in the HDX experiment are indicated by rectangular boxes below the hCFTR^{TS} construct sequence. The number in each box represents the change in the average deuterium uptake upon ADP binding across all 6 time points. Standard error and peptide ion charge state are also noted in parenthesis. The peptide boxes are colored based on deuterium uptake differences according to the colored key. For each peptide, the first 2 N-terminal amino acids were excluded from the analysis due to rapid back-exchange.

Figure S8

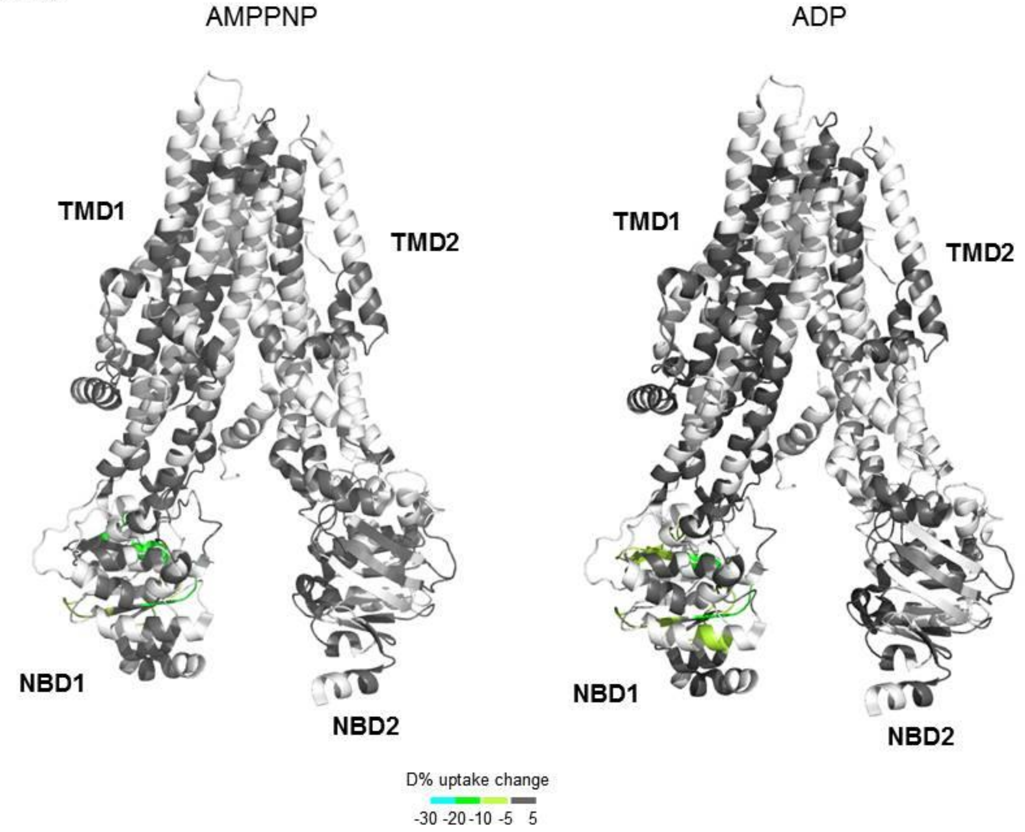

Figure S8: Comparison of the hCFTR^{TS} conformational changes upon AMPPNP (left) and ADP (right) binding. HDX perturbation data are mapped to the cryo-EM structure (PDB 5UAK). As shown in the key, a color gradient is used to represent the average deuterium uptake differences across all 6 time points between the apo and ligand bound states of hCFTR^{TS}. White indicates regions that were not detected for every replicate at every time point in the HDX experiments.

Figure S9

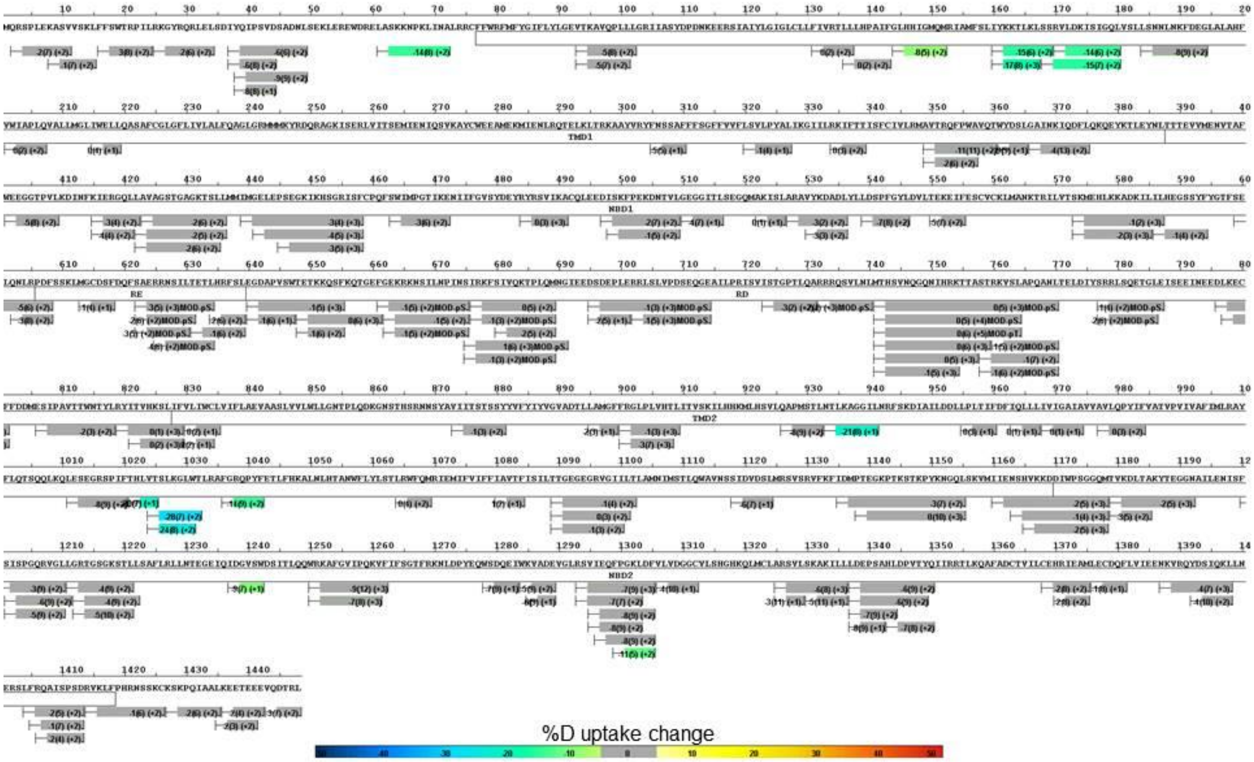

Figure S9: HDX perturbation data of Kalydeco binding mapped to hCFTR^{TS} sequence. Peptides analyzed in the HDX experiment are indicated by rectangular boxes below the hCFTR^{TS} construct sequence. The number in each box represents the change in the average deuterium uptake upon Kalydeco binding across all 6 time points. Standard error and peptide ion charge state are also noted in parenthesis. The peptide boxes are colored based on deuterium uptake differences according to the colored key. For each peptide, the first 2 N-terminal amino acids were excluded from the analysis due to rapid back-exchange.

Figure S10

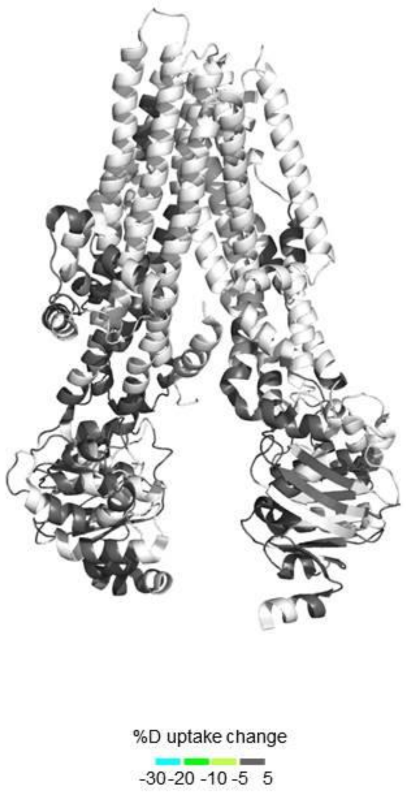

Figure S10: HDX perturbation data of Lumacaftor-bound dephosphorylated hCFTR^{TS}. As shown in the key, a color gradient is used to represent the average deuterium uptake differences across all 6 time points between the apo and Lumacaftor bound states of hCFTR^{TS}. No changes were detected by HDX. White indicates regions that were not detected for every replicate at every time point in the HDX experiments.

Figure S11

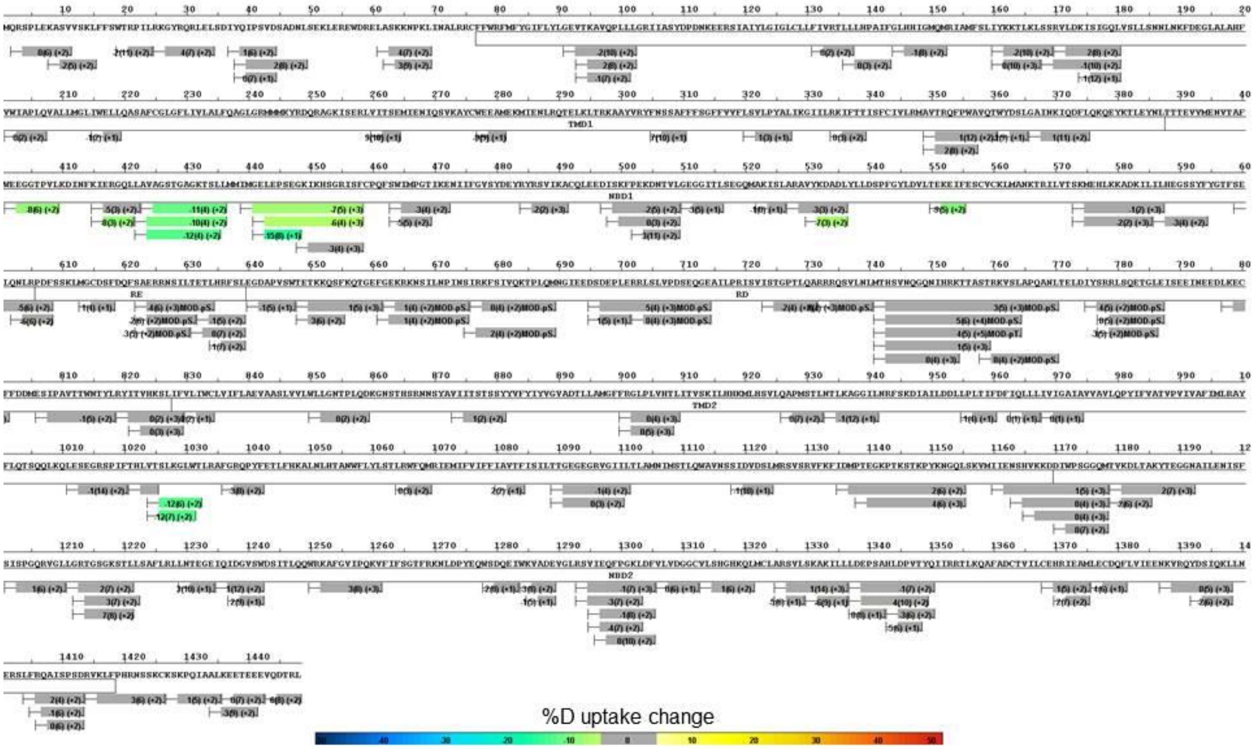

Figure S11: HDX perturbation data of AMPPNP + Kalydeco binding mapped to hCFTR^{TS} sequence. Peptides analyzed in the HDX experiment are indicated by rectangular boxes below the hCFTR^{TS} construct sequence. The number in each box represents the change in the average deuterium uptake upon AMPPNP + Kalydeco binding across all 6 time points. Standard error and peptide ion charge state are also noted in parenthesis. The peptide boxes are colored based on deuterium uptake differences according to the colored key. For each peptide, the first 2 N-terminal amino acids were excluded from the analysis due to rapid back-exchange.

Figure S12

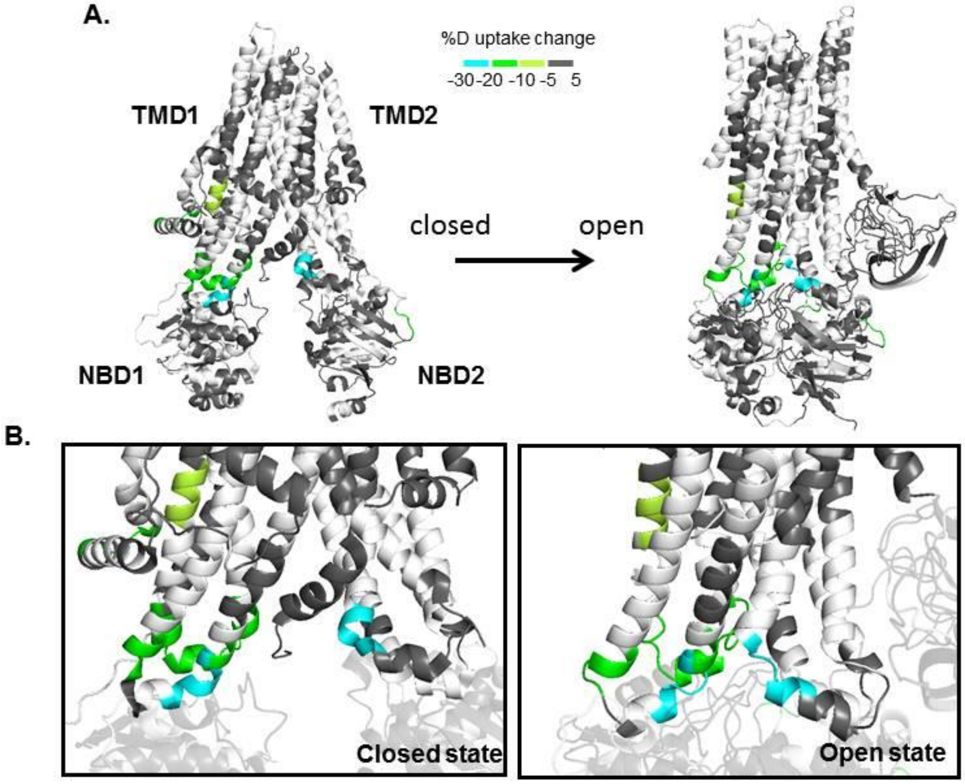

Figure S12: Structural comparison of regions protected in the presence of Kalydeco. (A) HDX perturbation data mapped to the closed-state cryo-EM structure (PDB 5UAK) *vs* the CFTR homology model (open state). As shown in the key a color gradient is used to represent the average deuterium uptake differences across all 6 time points between the apo and ligand bound states of $hCFTR^{TS}$. White indicates regions that were not consistently resolved in the HDX experiment. The open structure model shows the regions protected to exchange in ICL4 and ICL2 coming together. (B) The ICL regions are zoomed in to show the protected regions and new interactions made going from the closed state structure (left panel) to the open state model (right panel). Zoomed-in panels are shown in the same orientation as in (A), with NBDs in the closed-state structure as well as NBDs and the RD in the open model shown as partially transparent.

Figure S13

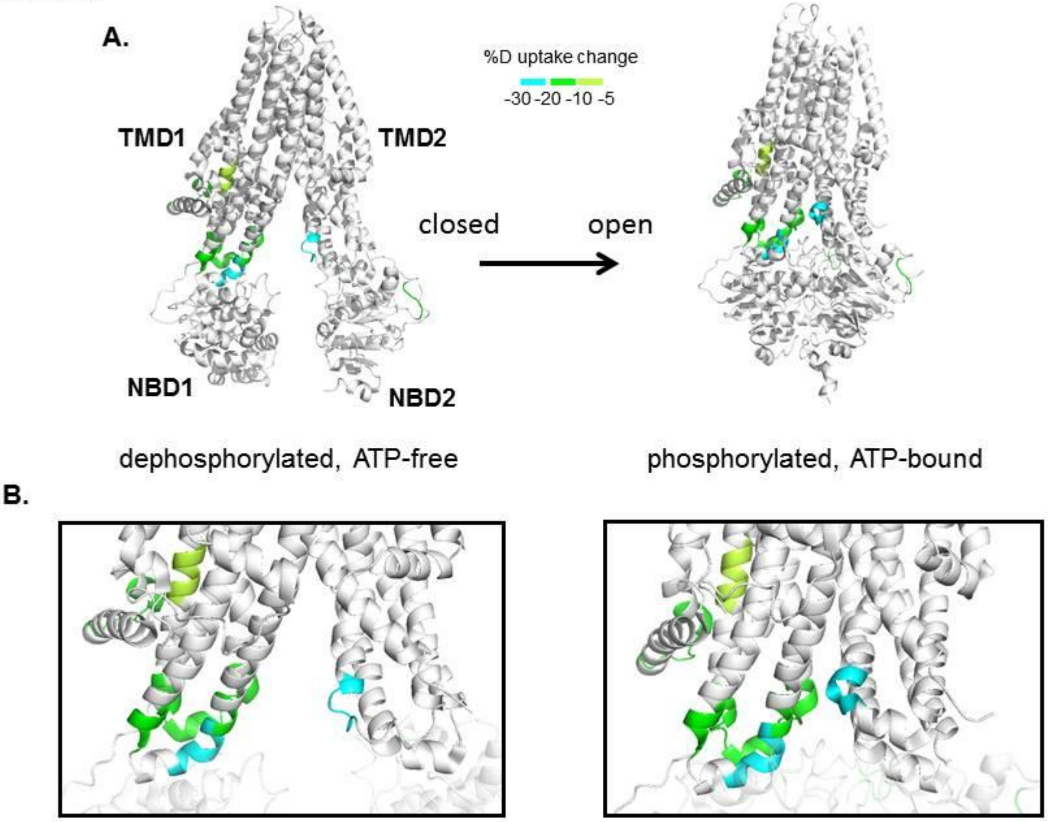

Figure S13: Structural comparison of regions protected in the presence of Kalydeco. (A) HDX perturbation data mapped to the dephosphorylated, ATP-free cryo-EM structure of zebrafish CFTR (PDB 5UAR) *vs* the phosphorylated, ATP-bound cryo-EM structure of zebrafish CFTR (PDB 5W81). As shown in the key a color gradient is used to represent the average deuterium uptake differences across all 6 time points between the apo and ligand bound states of $hCFTR^{TS}$. Only regions with more than 5% change in deuterium uptake are colored. The ATP-bound structure shows the regions protected to exchange in ICL4 and ICL2 coming together. (B) The ICL regions are zoomed in to show the protected regions and new interactions made going from the dephosphorylated, ATP-free structure (left panel) to the phosphorylated, ATP-bound structure (right panel). Zoomed-in panels are shown in the same orientation as in (A), with NBDs shown as partially transparent.

Figure S14

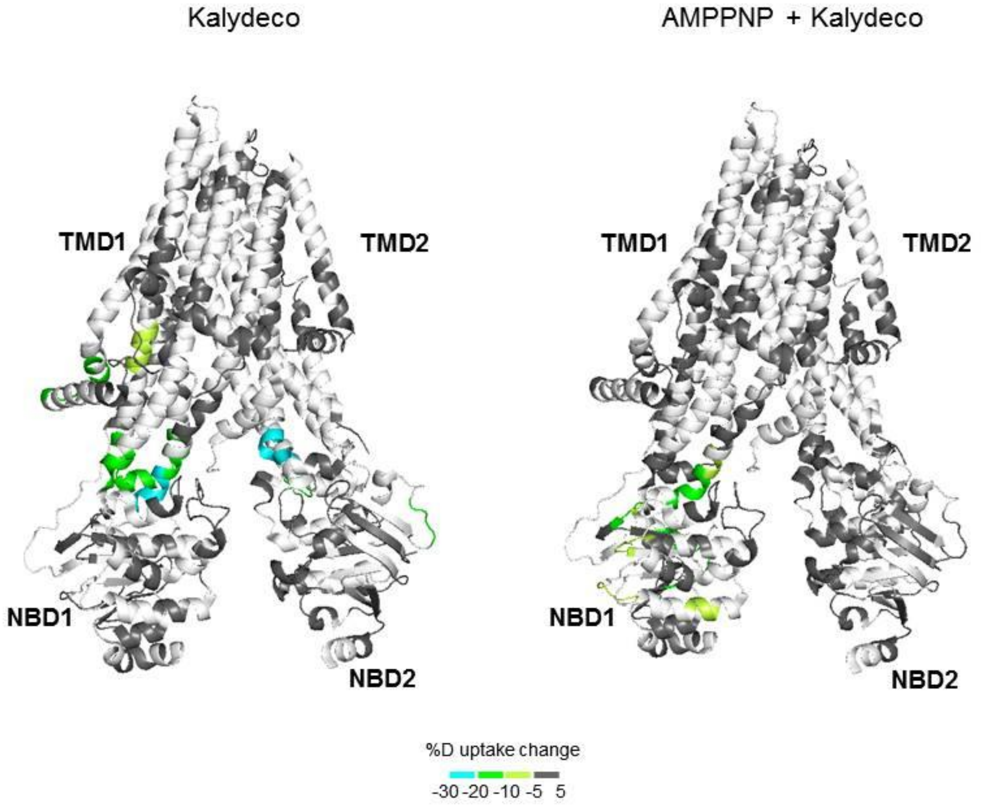

Figure S14: Comparison of the hCFTR[TS] conformational changes upon Kalydeco (left) and AMPPNP + Kalydeco (right) binding. HDX perturbation data are mapped to the cryo-EM structure (PDB 5UAK). As shown in the key, a color gradient is used to represent the average deuterium uptake differences across all 6 time points between the apo and ligand bound states of hCFTR[TS]. White indicates regions that were not consistently resolved in the HDX experiment.
